# Structure and Functionality of an Alkylated Li_*x*_Si_*y*_O_*z*_ Interphase for High-Energy Cathodes from DNP-ssNMR Spectroscopy

**DOI:** 10.1021/jacs.1c00215

**Published:** 2021-03-22

**Authors:** Shira Haber, Rosy, Arka Saha, Olga Brontvein, Raanan Carmieli, Arava Zohar, Malachi Noked, Michal Leskes

**Affiliations:** † Department of Materials and Interfaces, Weizmann Institute of Science, Rehovot, Israel 7610001; ‡ Department of Chemistry, Bar-Ilan University, Ramat Gan, Israel; § Bar-Ilan Institute of Nanotechnology and Advanced Materials, Ramat Gan, Israel; ∥ Department of Chemical Research Support, Weizmann Institute of Science, Rehovot, Israel 7610001; ¶ Department of Chemistry, Indian Institute of Technology BHU, Varanasi, India 221005

## Abstract

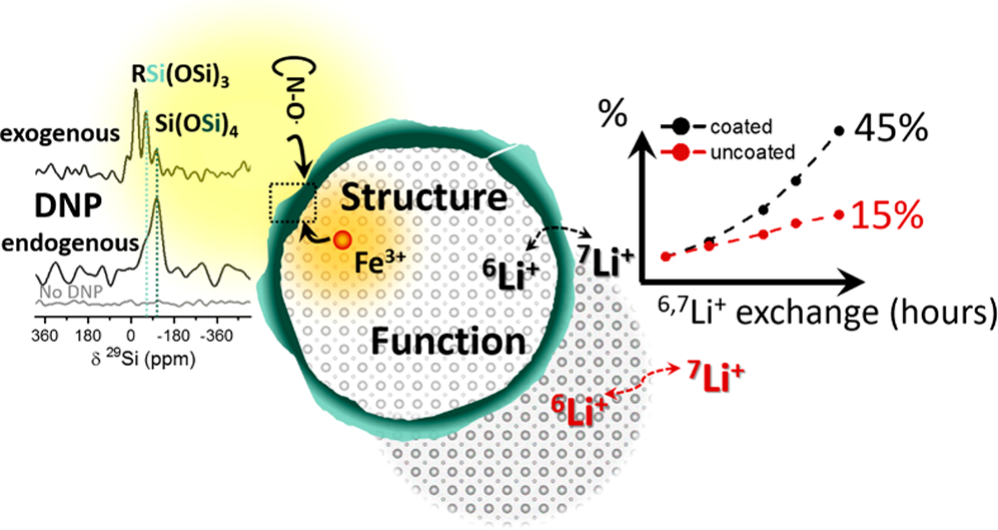

Degradation processes at the cathode–electrolyte interface are a major limitation in the development of high-energy lithium-ion rechargeable batteries. Deposition of protective thin coating layers on the surface of high-energy cathodes is a promising approach to control interfacial reactions. However, rational design of effective protection layers is limited by the scarcity of analytical tools that can probe thin, disordered, and heterogeneous phases. Here we propose a new structural approach based on solid-state nuclear magnetic resonance spectroscopy coupled with dynamic nuclear polarization (DNP) for characterizing thin coating layers. We demonstrate the approach on an efficient alkylated Li_*x*_Si_*y*_O_*z*_ coating layer. By utilizing different sources for DNP, exogenous from nitroxide biradicals and endogenous from paramagnetic metal ion dopants, we reveal the outer and inner surface layers of the deposited artificial interphase and construct a structural model for the coating. In addition, lithium isotope exchange experiments provide direct evidence for the function of the surface layer, shedding light on its role in the enhanced rate performance of coated cathodes. The presented methodology and results advance us in identifying the key properties of effective coatings and may enable rational design of protective and ion-conducting surface layers.

## 1 Introduction

The electrode–electrolyte interface plays a crucial role in the electrochemical performance of rechargeable batteries and in particular in lithium-ion battery (LIB) cells.^1,2^ Chemical and electrochemical reactions at the interface result in deposition of interphases which modify interfacial properties, such as electronic and ionic conductivity, and can thus completely block access to the electrode material. These reactions and their products have been thoroughly investigated on the anode side in LIBs, and great efforts were invested in identifying favorable conditions for the formation of a solid electrolyte interphase (SEI): interphases that will block electron transport and will prevent further reactivity with the electrolyte while still enabling efficient ion transport across the SEI.^2−4^ The cathode interface, on the other hand, which was initially thought to lead to negligible electrolyte decomposition, currently poses one of the key challenges in the development of high-energy LIB cells. This is due to the plethora of interfacial processes identified on the surface of high-energy cathodes, including electrolyte oxidation, metal ion dissolution, structural transformations, and oxygen evolution.^5,6^ Therefore, achieving control over cathode interfacial reactivity is essential for the development of high-energy cells.

A leading approach for gaining such control is through deposition of an artificial cathode–electrolyte interphase (CEI).^7,8^ Such CEI layers act as a passivating barrier between the cathode and the electrolyte and ideally should prevent chemical and structural degradation while maintaining ionic permeability. Despite the significant progress made in synthetic approaches to form highly efficient CEIs, far less is known about what are the properties that make a beneficial CEI. The ability to rationally design a permeable and passivating CEI is limited by the scarcity of analytical tools that can be used to probe thin (few nanometers), disordered, and heterogeneous layers. Furthermore, the ion transport properties of such interphases are mostly inferred from electrochemical impedance spectroscopy (EIS)^9−11^ and recently also modeling,^12−15^ and not from direct measurements of these phases. In recent years, the use of solid-state NMR (ssNMR) spectroscopy to characterize battery materials has increased considerably.^16,17^ The main advantage of this approach is its high chemical specificity, which combined with its short-range structural sensitivity, can be used to determine the chemical composition, three-dimensional arrangement, and evolution of phases at the atomic-molecular level. Moreover, it can be utilized to follow dynamic processes across a wide range of time scales, providing insight into chemical exchange and ion mobility.^18,19^

However, alongside the many advantages of ssNMR spectroscopy, its inherent low sensitivity, coupled with low abundance of many NMR-active nuclear isotopes, severely limits its broad applicability in the study of thin surface layers. Surface sensitivity can be gained by coupling ssNMR with dynamic nuclear polarization (DNP),^20−22^ a process in which the high electron spin polarization is transferred to the surrounding coupled nuclei by microwave (μwave) irradiation at specific transition frequencies. The development of highly efficient polarizing agents, based on nitroxide biradicals introduced into the sample of interest, results in 10–10^4^-fold increase in sensitivity. Such a boost in sensitivity enables the detection of otherwise extremely challenging surfaces and interfaces,^23^ including the SEI and interphases formed on various electrode materials.^24−26^ Another DNP approach is to transfer endogenous polarization from paramagnetic metal ion dopants.^27−31^ This approach has been successfully applied to gain NMR sensitivity in the bulk of oxides.^32−35^ To date, this approach has not been used to polarize interphases.

We have recently used the exogenous DNP approach to detect a novel surface treatment for high-energy cathodes based on a simple molecular layer deposition (MLD) process.^36^ MLD on the surface of Li and Mn-rich LiNi_*x*_Mn_*y*_Co_*x*_O_2_ (LMR-NMC) cathode, with alternating pulses of alkylsilyllithium and ozone as precursors, results in an efficient 2–5 nm thick alkylated Li_*x*_Si_*y*_O_*z*_ artificial CEI.^36^ Coated LMR-NMC cathodes have significantly improved electrochemical performance, especially enhanced rate performance, achieved through protection of the cathode and suppression of oxygen release during cycling. DNP-ssNMR provided insight into the chemical environments in the coating layer; however, it remains unknown why this specific coating is so efficient and what is the structural origin of its enhanced rate performance.

**Figure 1:**
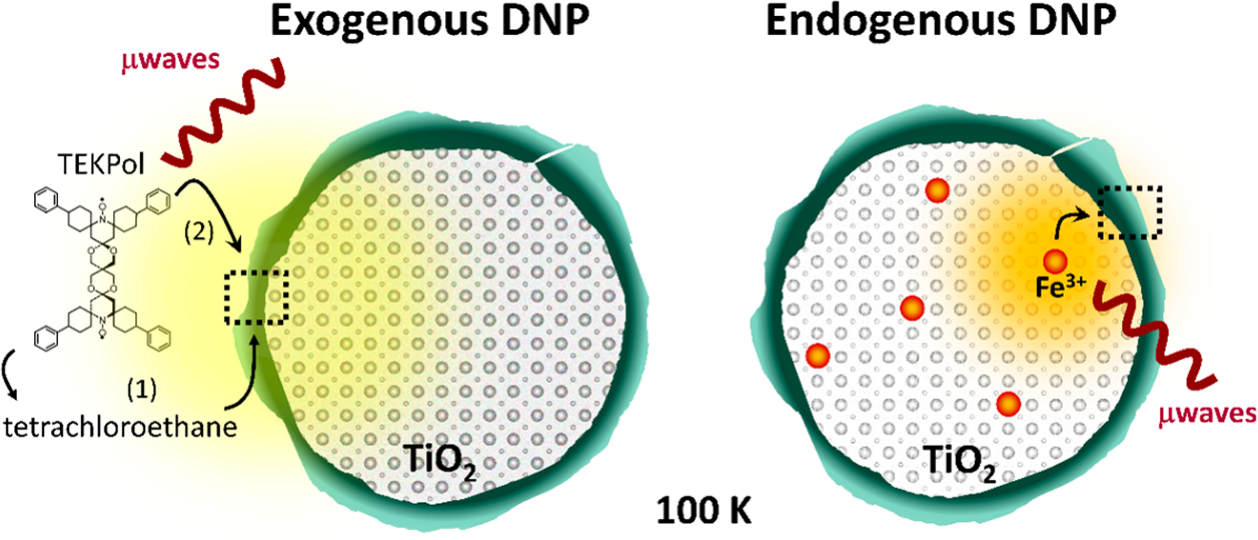
Schematic representation of the DNP approaches employed in this work. For exogenous DNP, the coated sample was wetted with a nitroxide solution (16 mM TEKPol in tetrachloroethane) and cooled to 100 K in the DNP probe. Irradiation with microwaves leads to polarization transfer from the radicals to the sample (1) indirectly, through the ^1^H in the solvent and then to the sample, or (2) directly, from the radicals to the nuclei in the surface layer. Endogenous DNP was performed with paramagnetic Fe(III) dopants introduced into coated TiO_2_ particles. Irradiation with microwaves at 100 K leads to direct polarization transfer from the Fe(III) d electrons to the nuclei in the inner surface of the coating layer.

Here we provide a detailed analysis of the composition and structure of this efficient artificial CEI. We first present results on a model system, TiO_2_, that went through identical MLD treatment as the LMR-NMC. We chose this substrate as it does not contain lithium and it is diamagnetic (excluding the effect of defects) and thereby does not interfere with detection of the CEI. We combine the exogenous DNP approach, where surface polarization is obtained from a solution of TEKPol radicals wetting the sample, with endogenous DNP based on polarization transfer from Fe(III) dopants in the bulk of the coated particles to their surface. Exogenous DNP provides high sensitivity which enables obtaining a detailed compositional map of the CEI. Figure 1 depicts the two approaches and the polarization transfer pathways of exogenous and endogenous DNP. We then show that by comparing spectra obtained by polarizing the nuclei in the thin surface coating from inside (from paramagnetic metal ion dopants) and outside (from nitroxide radicals), we can probe the interface between the coating and the electrode and determine the spatial arrangement of the CEI.

Finally, we perform isotope exchange experiments on LMR-NMC and coated TiO_2_, where we follow the spontaneous exchange process of the two NMR-active isotopes of lithium, ^6,7^Li. By tracking the exchange process, we gain direct insight into the functionality of the CEI and its role in ion transport across the electrode–electrolyte interface.

## 2 Materials and Methods

### 2.1 Materials

The synthesis of spherical TiO_2_ particles followed the previously reported procedure.^36^

Fe-doped TiO_2_ (Fe–TiO_2_) particles were synthesized in two consecutive steps: (i) synthesis of amorphous spherical Fe doped titanium glycolate through precipitation and (ii) high-temperature annealing/calcination of the resulting Fe–TiO_2_.^37^ For the first step, 50 mL of ethylene glycol (EG, Sigma-Aldrich) was put in a 100 mL conical flask and purged with nitrogen for 30 min under continuous stirring to remove dissolved oxygen. Next, 4.725 mg (0.5 mol %) of Fe(NO_3_)_2_·9H_2_O (Sigma-Aldrich) was added to the EG solution followed by the dropwise addition of 0.8 mL of tetrabutoxytitanium (Sigma-Aldrich). After 15 min of stirring, the resulting solution was sealed with parafilm and stirred for another 8–10 h at room temperature. The obtained transparent mixture was then added into 200 mL of acetone, followed by addition of 2 mL of water. The mixture was then sealed with parafilm and stirred for another 1–2 h. The white precipitate obtained, composed of spherical Fe-doped titanium glycolate, was collected by centrifugation, washed with ethanol three times, and dried overnight at 60 °C. Further annealing of the powder at 350 °C for 3 h, ramp rate of 1 °C/min, led to spherical Fe–TiO_2_ particles.

Li_*x*_Si_*y*_O_*z*_ molecular layer deposition (MLD) treatment of TiO_2_, Fe–TiO_2_, and LMR-NMC particles was identical with the previously reported procedure.^36^

### 2.2 Characterization Techniques

The crystal structure and purity of lithium metasilicate were determined by powder X-ray diffraction (PXRD) measurements on a TTRAX-III, Rigaku diffractometer equipped with a rotating Cu anode operating at 50 kV and 200 mA. The 2θ scanning range was 5°–80° with a scan rate of 1 °C/min. Phase analysis was performed using JADE 2010 software. PXRD for Fe–TiO_2_ was performed on a Bruker D8 Advanced X-ray diffractometer, using Cu Kα radiation in the range of 2θ from 10° to 80°, with a scan rate of ≈0.0194 °C/min.

STEM examinations were performed with a Thermo Fisher Scientific Titan Themis Z transmission electron microscope operated at 200 kV, equipped with Super-X large solid angle X-ray detector for EDS. Images and EDS maps were collected from various particles in the samples.

EPR measurements were performed on a Bruker ELEXYS E-580 spectrometer operating at Q-band (35 GHz) fitted with a Q-band resonator (EN5107-D2) at the temperature of 50 K. The temperature was controlled by a Bruker FlexLine cryogen free VT system ER4118HV-CF5-H. Field-sweep echo-detected (FSED) EPR spectra were recorded by using the two-pulse echo sequence (π/2–*t*–π–*t*–echo) where the echo intensity is measured as a function of the magnetic field. The microwave pulse lengths π/2 and π were 14 and 28 ns, respectively, and the time interval between the pulses, *t*, was 110 ns.

### 2.3 Electrochemistry

Coin cells (type 2032) were assembled in an Ar-filled glovebox with ^6^Li metal (Sigma-Aldrich) as anode and uncoated 0.35Li_2_MnO_3_·0.65LiNi_0.35_Mn_0.45_Co_0.20_O_2_ (LMR-NMC) powder or Li_*x*_Si_*y*_O_*z*_-LMR-NMC powder cathodes (85% active mass 15% carbon black C65, Imerys). A borosilicate separator (Sigma-Aldrich) on top of a Celgard separator was used between the two electrodes with 7 drops of LiPF_6_ 1 M in 50:50 dimethyl carbonate (DMC):ethylene carbonate (EC) (LP30) electrolyte (Solvionic). Currents for C-rates were calculated with the specific capacity of LMR-NMC as 250 mAh g^–1^. The electrochemical measurements were performed by using BCS-805 battery cycler and Bio-Logic VMP3 cycler (Biologic Science Instruments) in a potential window of 2.0–4.7 V. The first charge–discharge cycle was performed at C/15 with 4.7 V as the cutoff voltage. An additional four cycles were performed at C/10 with upper cutoff voltage of 4.6 V. Batteries were disassembled in the glovebox; the cathode powder was scraped, washed with anhydrous DMC (Sigma-Aldrich), and dried overnight in the prechamber. Electrochemical impedance spectra (EIS) of the uncoated and coated LMR-NMC electrodes were recorded after five cycles, with an amplitude of 10 mV and a frequency range of 1 MHz–5 mHz. Each sample was tested after 5 h potentiostatic steps to measure the electrode at steady-state conditions^38^ by using a Bio-Logic VMP3 cycler.

### 2.4 DNP and NMR Sample Preparation

For exogenous DNP measurements, coated TiO_2_ particles were dried overnight in a vacuum oven at 100 °C and packed into a 3.2 sapphire rotor in an Ar-filled glovebox. A few microliters of the radical solution (16 mM TEKPol (Cortecnet) in tetrachloroethane (Sigma-Aldrich)) was added to the rotor resulting in a moist powder. The rotor was closed with a Teflon plug and zirconia cap and inserted into the low-temperature probe kept at about 100 K. The weight and details of the samples measured are provided in Table S1.

For endogenous DNP measurements, Fe–TiO_2_ samples were dried overnight in a vacuum oven at 100 °C; between 4 and 22 mg were packed into a 3.2 sapphire rotor in an Ar-filled glovebox. The rotor was closed with a Teflon plug and zirconia cap and inserted into the low-temperature probe kept at about 100 K.

Samples for ^6,7^Li exchange experiments on uncoated and coated LMR-NMC were prepared by placing 5–8 mg of ^6^Li-enriched uncoated LMR-NMC and Li_*x*_Si_*y*_O_*z*_-LMR-NMC in Eppendorf tubes and adding 50 μL of LP30 to cover the powder. After 10, 20, 45, 60, and 80 h, LP30 was extracted; samples were washed three times with anhydrous DMC and dried overnight in the glovebox prechamber. 2.5–5 mg of the different samples were packed in 1.3 mm zirconia rotors.

For detecting the ^6,7^Li exchange in the coating layer, two samples of dry Li_*x*_Si_*y*_O_*z*_–TiO_2_ (∼48 mg) were placed in 50 μL of 0.025 M ^6^LiPF_6_ solution and 0.025 M ^7^LiPF_6_ solution for 45 h. The powders were then washed three times with anhydrous DMC to remove residual electrolyte and dried overnight in the glovebox prechamber. The electrolyte for these experiments was prepared by dissolving the appropriate amount of ^6,7^LiPF_6_ (Sigma-Aldrich) in 1:1 weight ratio of EC (Alfa Aesar) and DMC (Sigma-Aldrich).

### 2.5 ssNMR Experiments

Solid-state NMR experiments were performed on 9.4 T Bruker Avance III and Avance Neo 400 MHz wide bore spectrometers. Samples were packed into rotors with an outer diameter of 1.3 or 4 mm for magic-angle spinning experiments with sample spinning at 50 and 10 kHz, respectively. Details on specific samples and experimental parameters used are given in Tables S1–S4. ^7^Li spectra were referenced to LiF at −1 ppm, and ^6^Li experiments were referenced to Ni-doped lithium titanate at 0 ppm. Quantification of spectra was done with the TOPSPIN program.

### 2.6 Magic-Angle Spinning DNP Experiments

DNP experiments were performed on a Bruker 9.4 T Avance-Neo spectrometer equipped with a sweep coil and a 263 GHz gyrotron system. We used 3.2 mm triple and double resonance low-temperature DNP probes for the experiments at magic-angle spinning of 10 kHz. All experiments were performed at about 100 K, with sample temperature of about 99 and 105 K without and with microwave irradiation, respectively. All spectra were acquired after the sample temperature was stable. Longitudinal relaxation, *T*_1_, and polarization buildup time with microwave irradiation, *T*_bu_, were measured with the saturation recovery pulse sequence by using a train (50 repetitions) of short pulses separated by 1 ms delays for saturation. ^1^H relaxation experiments were analyzed using TOPSPIN and fitted with ORIGIN software.

^1^H experiments were acquired by using a rotor synchronized Hahn echo sequence. ^1^H–^29^Si and ^1^H–^7^Li cross-polarization magic-angle spinning experiments were performed with a ramp on the ^1^H channel. ^1^H–^13^C cross-polarization magic-angle spinning experiments were performed by using swept-frequency two-pulse phase modulation (sw_f_TPPM)^39^^1^H decoupling. Direct detection of ^29^Si was performed by using the Carr–Purcell–Meiboom–Gill (CPMG) technique.

^7^Li/^1^H–^29^Si{^7^Li} cross-polarization REDOR experiments were implemented in a pseudo-two-dimensional manner following ^7^Li–^29^Si cross-polarization for lithium metasilicate and ^1^H–^29^Si cross-polarization for lithium silicate-coated TiO_2_ samples. All experiments preceded by a saturation period achieved by using a train (20–50 repetitions) of short pulses separated by 1 ms delays.

For following isotope exchange in the coating, exogenous DNP was employed with direct ^6^Li and indirect polarization through ^1^H–^6^Li cross-polarization magic-angle spinning. ^1^H and ^6^Li relaxation experiments were analyzed using TOPSPIN and fitted with ORIGIN software. ^1^H and ^13^C were referenced to tetrachloroethane at 6.4 and 74 ppm, respectively, and the ^29^Si chemical shift to kaolinite at −91.5 ppm. ^7^Li spectra were referenced to LiF at −1 ppm. Additional details on specific samples and experimental parameters used are given in Tables S1–S4.

## 3 Results and Discussion

### 3.1 CEI Composition and Structure

Characterization of the coating was first attempted on the Li_*x*_Si_*y*_O_*z*_-coated LMR-NMC powder. Detection of ^7^Li environments in the coating proved challenging due to significant spectral overlap with the dominant lithium resonances from the bulk of the cathode (Figure S1). To avoid interference from the bulk, experiments were performed on Li_*x*_Si_*y*_O_*z*_-coated TiO_2_. Room temperature ^7^Li MAS experiments resulted in a poor signal-to-noise ratio in ^7^Li spectra, even after 24 h of acquisition from ∼100 mg sample (Figure S2). Cross-polarization experiments are commonly used to increase the sensitivity of low abundance and/or low sensitivity nuclei by transferring the polarization from ^1^H nuclei with large magnetic moment and high abundance to nuclei in close proximity (a few angstroms). However, the limited thickness of the coating also prevented detection of ^29^Si resonance through ^1^H–^29^Si cross-polarization experiments. Thus, to gain sensitivity in probing the coating without interference from bulk signals, the coating was further characterized through magic-angle spinning DNP measurements on the Li_*x*_Si_*y*_O_*z*_-coated TiO_2_ samples. Two DNP approaches were used, differing in the location of the polarization source (schematically described in Figure 1), to provide sensitivity to different areas of the coating.

#### 3.1.1 Exogenous DNP

**Figure 2:**
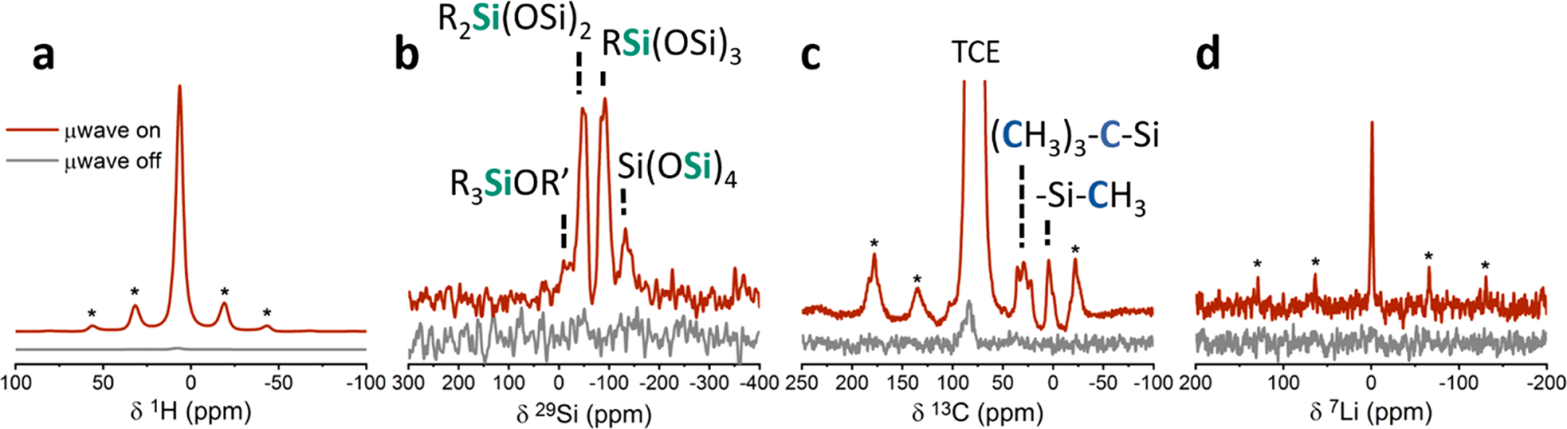
(a) ^1^H rotor synchronized Hahn echo spectra of the Li_*x*_Si_*y*_O_*z*_–TiO_2_ sample acquired with and without microwave irradiation by using a polarization time of 45 s and 2 scans. (b) ^29^Si spectra acquired with and without microwaves by using indirect DNP with ^1^H–^29^Si cross-polarization with polarization time of 6 s, 3072 scans, and 2 ms contact time. (c) ^13^C spectra acquired with indirect DNP by ^1^H–^13^C cross-polarization with polarization time of 5 s, 128 scans, and 1 ms contact time. TCE marks the ^13^C resonance of the tetrachloroethane solvent. (d) Indirect DNP spectra of ^1^H–^7^Li cross-polarization acquired with polarization time of 10 s, 256 scans, and 1 ms contact time. All experiments were performed at 100 K with 10 kHz spinning speed. Spinning sidebands are marked with an asterisk.

Exogenous DNP experiments were performed on coated TiO_2_ particles by wetting the sample with 16 mM TEKPol in tetrachloroethane solution following the common approach for DNP surface-enhanced NMR spectroscopy.^20^ The polarization of the ^1^H of tetrachloroethane, with 250-fold enhancement from DNP (Figure 2a), was transferred to the ^29^Si, ^13^C, and ^7^Li species in the lithium silicate thin surface layer through cross-polarization, enabling the assignment of the local environments in the CEI. In the ^1^H–^29^Si cross-polarization spectrum (Figure 2b), four ^29^Si environments were detected and assigned: double and monoalkylated silica groups resonating at −20 and −60 ppm, respectively,^40^ an amorphous silica environment at −110 ppm,^41^ and a triple alkylated silicon group at 17 ppm (R′ is assigned to −H or an alkyl group).^40,42^ X-ray photoelectron spectroscopy (XPS) has previously shown the silicon environments found on the lithium silicate thin film.^36^ Silica, double, and monoalkylated groups were detected by XPS and correlate well with the findings of DNP-ssNMR. The triple alkylated silicon group, found in the ^29^Si spectrum, is of lower intensity than the other silicon environments and may be below the detectability of the XPS technique.

Indirect DNP from ^1^H nuclei to ^13^C nuclei, shown in Figure 2c, revealed four carbon groups that are assigned to the *tert*-butyl environment (20, 27, and 37 ppm) and methyl groups (1.85 ppm)^42,43^ originating from the single source MLD precursor material.^36^ A broad ^7^Li resonance, centered at 0 ppm,^44^ was identified through polarization transfer from ^1^H nuclei (Figure 2d), suggesting that lithium sites are accessible to the solvent and are found on the outer surface layer of the lithium silicate coating. Table 1 summarizes the various environments found in the lithium silicate coating layer, with their chemical shifts.

**Table 1:** Lithium Silicate Compositional Environments and Chemical Shift Assignments

nucleus	chemical shift (ppm)	assignment	ref
^1^H	6.4	tetrachloroethane	
^29^Si	17	R_3_-SiOR′^a^^,^^b^	(40, 42)
	–20	R_2_-Si(OR)_2_^a^	(40)
	–60	R-Si(OR)_3_^a^	(40)
	–110	SiO_*x*_	(41)
^13^C	20, 27, and 37	(CH_3_)_3_-CH-	(42, 43)
	1.85	CH_3_	(42, 43)
^7^Li	0	interfacial lithium	(44)

a R is assigned to an alkyl group.

b R′ is assigned to −H or an alkyl group.

**Figure 3:**
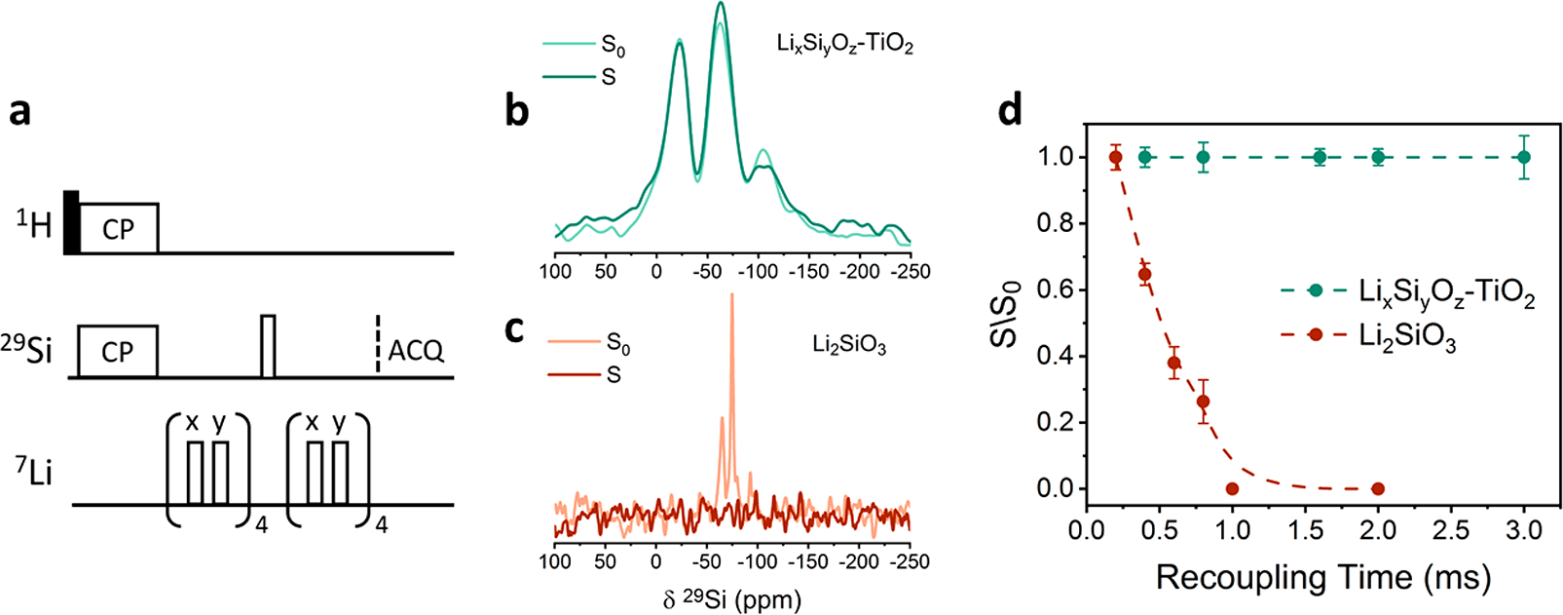
(a) Pulse sequence for rotational echo double resonance (REDOR) experiment. (b) ^1^H–^29^Si{^7^Li} cross-polarization REDOR DNP experiment performed on the lithium silicate-coated TiO_2_ sample with polarization time of 5 s, 8800 scans, 2 ms contact time, and 3 ms recoupling time. (c) ^7^Li–^29^Si{^7^Li} cross-polarization REDOR slices acquired from lithium metasilicate with relaxation delay of 90 s, 48 scans, 4 ms contact time, and 2 ms recoupling time. (d) Normalized integrated intensity of ^1^H/^7^Li–^29^Si{^7^Li} cross-polarization REDOR experiments as a function of the recoupling time for lithium silicate-coated TiO_2_ (green) and for lithium metasilicate for peak at −75 ppm (dark red). Experiments were performed at 100 K with 10 kHz spinning speed.

To determine whether the detected Li and Si environments are found in the same phase in the thin coating layer, distance measurement experiments were performed with the ^1^H–^29^Si{^7^Li} cross-polarization rotational echo double resonance (cross-polarization REDOR, Figure 3a) technique,^45^ enabled by the sensitivity gained from DNP. This technique reintroduces the dipolar coupling between the lithium and silicon nuclei, which are otherwise averaged out by magic-angle spinning. Measurements of the signal decay with increased dipolar recoupling time provide information about the proximity between Si and Li environments. To account for the decay due to ^29^Si transverse relaxation, the signal with recoupling pulses on the ^7^Li (S) is divided by the reference signal without recoupling pulses (S_0_). In Figure 3b, the S and S_0_ spectra, acquired following a dephasing period of 3 ms, are compared for the lithium silicate-coated TiO_2_, showing that there is no significant difference between the spectra (above the noise level). These results were compared with the ^7^Li–^29^Si{^7^Li} cross-polarization REDOR experiment performed on a model compound, lithium metasilicate, having close Si and Li pairs within 3 Å (Figure S3a). The resonance of the main ^29^Si environment in the lithium metasilicate, at −75 ppm^46,47^ (resonances at −65 and −100 ppm are assigned to Li_4_SiO_4_ and SiO_2_ impurities, respectively,^47^Figure S4), completely decayed in <1 ms recoupling time (Figure 3c). In Figure 3d, the dephasing curves for the Li_*x*_Si_*y*_O_*z*_–TiO_2_ and the model compound are compared. Numerical simulations performed with SPINEVOLUTION^48^ (see Figure S3b) suggest that the Li–Si pairs in the coating have to be more than 6 Å apart; otherwise, they would show measurable decay. Thus, we can conclude from these REDOR experiments that the direct bonds between Li and Si atoms in the precursor dissociate during the MLD process, leading to Li sites being at least 6 Å removed from Si sites, most likely in separate phases.

The DNP surface-enhanced NMR spectroscopy approach provides excellent sensitivity to the surface of the sample. The extent of polarization transfer across the surface and toward the bulk typically depends on the ability of the nuclei involved to propagate the polarization through efficient spin diffusion.^21,49^ In the present case, we employed indirect polarization through cross-polarization from ^1^H nuclei. Thus, we can enhance resonances (of ^29^Si, ^13^C, and ^7^Li) that are directly accessible to the solvent or have proton environments in close proximity (which can get polarized by spin diffusion from the polarized solvent or directly from the nitroxide radicals). Based on this, the precursor used, and the nature of the MLD process, the alkylated species are found at the outer interface of the coating.

#### 3.1.2 Endogenous DNP

**Figure 4:**
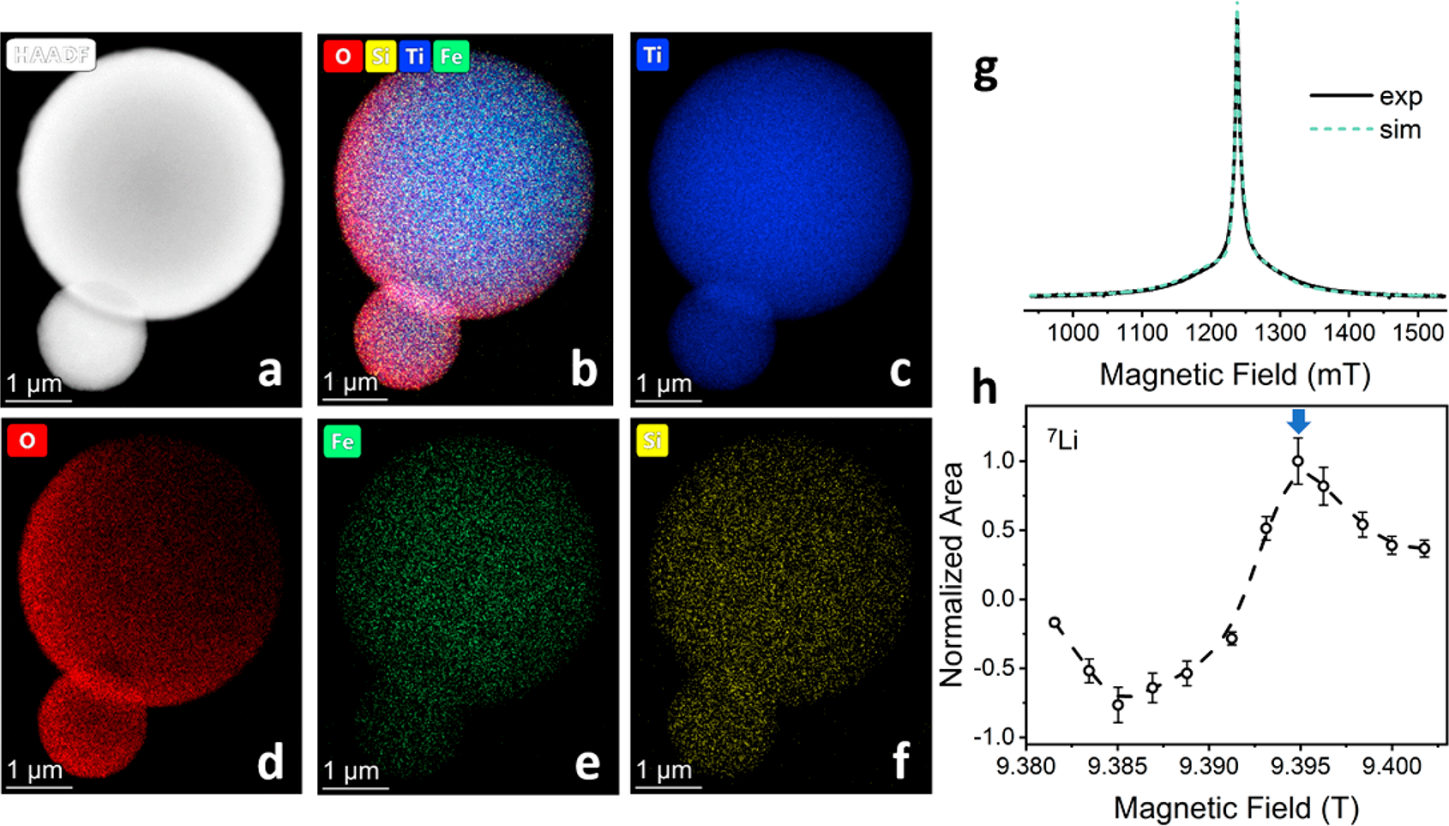
(a–f) HAADF-STEM analysis of Li_*x*_Si_*y*_O_*z*_-coated Fe-TiO_2_ particles showing the elemental distribution of (c) Ti, (d) O, (e) Fe, and (f) Si. (g) Field sweep echo detected Q-band of Fe–TiO_2_ (black) and fitted simulation (green) calculated with *S* = 5/2, *g* = 1.99, *D* = 1500 MHz, and *E* = 0. (h) DNP sweep profile acquired for ^7^Li direct polarization with a buildup time of 20 s and 4 scans for the lithium silicate-coated Fe-TiO_2_ sample. The field was set to 9.395 T (blue arrow).

To assess the uniformity of the lithium ions distribution throughout the coating layer, as well as obtain insight into the composition of the interface of the coating with the substrate, we employed the endogenous DNP approach. To this end, micrometer-sized TiO_2_ particles were doped with Fe(III)^35^ at a nominal concentration of 0.5% mol (60 mM) (XRD of the synthesized anatase phase is shown in Figure S5) and coated with the Li_*x*_Si_*y*_O_*z*_ surface layer. Figure 4a shows the high-angle annular dark-field images collected in the scanning transmission electron microscope (HAADF-STEM) from the Fe–TiO_2_-coated powder. The HAADF-STEM analysis (shown in Figure 4b–f) confirmed the homogeneous distribution of the doped iron (atomic fraction of 0.6% ± 0.14%) and the coated silicon (atomic fraction of 0.49% ± 0.13%) (Figures 4e and 4f, respectively). Figure 4g displays the field sweep echo detected EPR spectrum for Fe–TiO_2_, acquired at 34.2 GHz (Q-band) at 50 K. The spectrum displayed a typical powder pattern of high spin electron species and was fitted with EASYSPIN^50^ to a single site with *S* = 5/2 broadened by zero field splitting with *D* of 1500 MHz and a similar D strain. A similar pattern was observed for Fe(III) doped in Li_4_Ti_5_O_12_ anode,^35^ further confirming the incorporation of the Fe(III) dopants in the TiO_2_ particles.

The coated Fe–TiO_2_ particles were then studied with magic-angle spinning DNP at 100 K. First, a DNP sweep profile was acquired (Figure 4h) by measuring the signal intensity of ^7^Li resonance in the coating (with microwave irradiation) as a function of the magnetic field. The field dependence displayed the typical positive and negative signal enhancement lobes, separated by about twice the Larmor frequency of ^7^Li, suggesting that the DNP mechanism for polarization transfer is the solid effect.^51^ The magnetic field was then set to the position that gave the highest signal intensity (marked by a blue arrow in Figure 4h).

**Figure 5:**
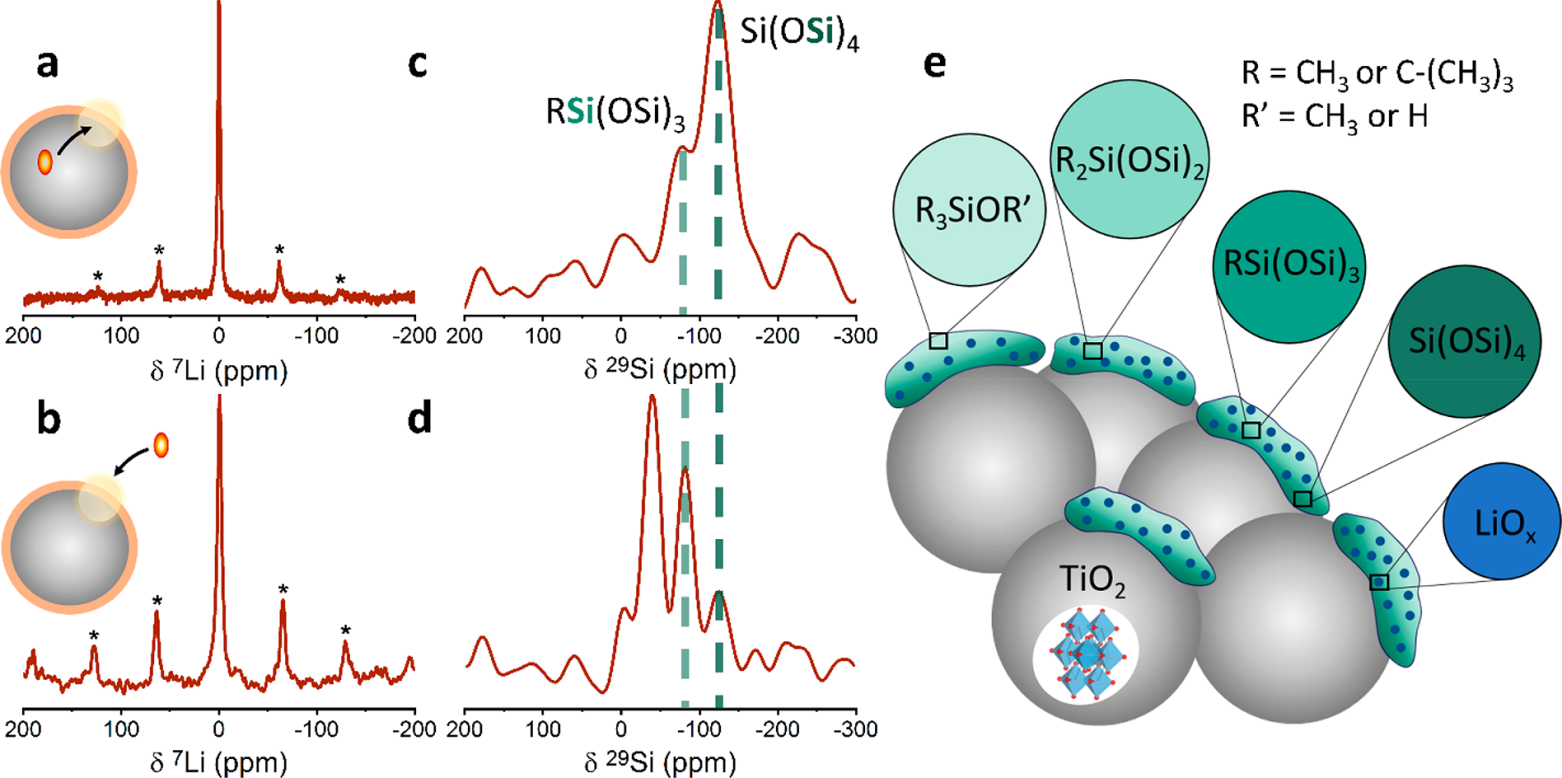
Top spectra: direct polarization via endogenous DNP from the unpaired electrons of the iron dopant (inset: polarization source represented as red ellipse) to (a) ^7^Li nuclei, acquired with polarization time of 33 s and 128 scans and (c) ^29^Si nuclei using CPMG detection, acquired with polarization time of 300 s and 126 scans. Bottom spectra: direct polarization via exogenous DNP from the unpaired electrons of the nitroxide solution (inset) to the (b) ^7^Li nuclei, acquired with polarization time of 100 s and 8 scans and (d) ^29^Si nuclei by using CPMG detection, acquired with polarization time of 120 s and 192 scans. Experiments were performed at 100 K with 10 kHz spinning speed. Monoalkylated silica and silica groups are marked with light green and dark green dotted lines, respectively. (e) A structural model of the Li_*x*_Si_*y*_O_*z*_ coating layer showing the various silicon environments in different shades of green. Uniformly distributed LiO_*x*_ is shown in blue.

At the optimal magnetic field for polarizing ^7^Li from the iron dopant, with microwave irradiation, the polarization is transferred from the Fe(III) d electrons to the surrounding coupled nuclei. This enabled examination of the environments in the surface layer through direct polarization transfer to ^7^Li and ^29^Si as shown in Figures 5a and 5c, respectively. At the optimal position, a polarization buildup time of 23 s was measured for ^7^Li and an enhancement factor of 8 was obtained for the lithium nuclei, at steady state. We note that the enhancement can probably be increased by optimizing the Fe(III) content. ^29^Si detection was also enabled by polarization transfer from Fe(III) (without optimization of the field, which would probably result in higher sensitivity) by using CPMG detection with a polarization time of 300 s. Two silicon environments were detected in the CPMG measurements which can be assigned to monoalkylated silica and SiO_*x*_ groups (resonating at −60 and −110 ppm).

Recently, we have shown that direct polarization transfer in the bulk of Fe(III)-doped Li_4_Ti_5_O_12_ is distance independent in cases where the dominant nuclear relaxation mechanism is the paramagnetic dopant.^33^ In the current system, because of the presence of strong dipole moments of ^1^H and ^7^Li in the coating and overall heterogeneity and disorder in the coating layer, it is unlikely that the Fe(III) dopants in the bulk are the only source of relaxation. Thus, we assume direct polarization to be limited in this case to a few atomic layers from the doped TiO_2_ surface. The fact that lithium and silicon nuclei could be detected by endogenous DNP indicates that there are lithium and silicon environments at the inner surface layer of the coating.

By comparing the resonances detected when polarization is transferred directly from exogenous nitroxide radicals to those detected when polarization is transferred endogenously from the Fe(III) dopants, we can gain structural insight into the arrangement of the phases on the surface. Figures 5b and 5d show the direct polarization of ^7^Li and ^29^Si environments, respectively, acquired via polarization transfer from the nitroxide solution to the coupled nuclei. Comparison of the spectra collected with endogenous DNP (upper Figures 5a and 5c) and with exogenous DNP (lower Figures 5b and 5d) suggests that (i) lithium ions are distributed uniformly throughout the coating layer as they can be detected using both polarization sources and (ii) monoalkylated silicon and SiO_*x*_ groups are at the inner layer closer to the TiO_2_, as they are revealed with endogenous DNP and are less exposed to the nitroxide solution, resulting in relatively low intensity with exogenous DNP. We note the comparison is between the chemical environments detected and not the relative intensities in the spectra since different samples and sources for polarization were employed. Nonetheless, as both sets of experiments were performed by direct polarization which requires the nuclei to be close to the polarization source, they provide structural information about the coating layer.

On the basis of these multinuclear exogenous and endogenous DNP-NMR results, we propose a structural model for the coating layer shown in Figure 5e. The CEI is composed of a thin, open interface of amorphous silica, terminated with siloxanes and alkylated (*tert*-butyl and methyl) silicon groups. Lithium forms separate domains from the silicon that are uniformly dispersed throughout the coating layer.

### 3.2 Li Ion Mobility across the CEI

**Figure 6:**
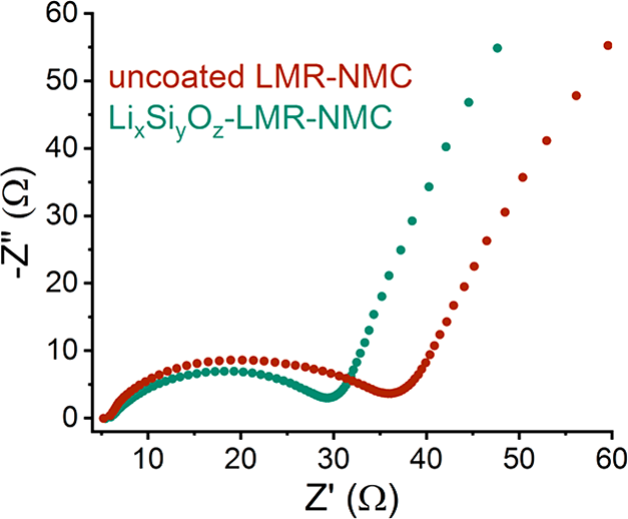
EIS Nyquist plots measured at the discharged state of the uncoated and lithium silicate coated LMR-NMC electrodes vs Li metal after five cycles.

We now turn to determine the functionality of the artificial CEI and its role in lithium ion transport across the electrode interface. Previously, we observed improved electrochemical performance, in particular rate performance, for Li_*x*_Si_*y*_O_*z*_-coated LMR-NMC compared to the uncoated cathode.^36^ As galvanostatic cycling tests are performed at the cell level, it is difficult to isolate specific factors leading to the improved performance. EIS measurements were performed to determine the effect of the coating layer on interfacial transport properties. Figure 6 portrays the EIS Nyquist plot measured for the uncoated and coated cathodes following five galvanostatic cycles vs Li metal (Figure S6). Because these measurements were performed in a two-electrode cell, it is not possible to assign the various features in the EIS spectra to one of the electrodes; therefore, only a qualitative discussion can be done, comparing the different electrochemical cells. The spectra show that the semicircle in the high–medium frequency (which can be attributed to charge transfer and surface film resistances^52,53^) of the lithium silicate-coated sample is smaller than that of the uncoated sample. This suggests that Li ion migration through the electrode interface is improved with the lithium silicate surface treatment. We cannot rule out the possibility that the higher resistance of the uncoated cathode is a result of CEI formation. CEI formation on LMR-NMC occurs upon cathode soaking in the electrolyte, and an ∼12 nm thick CEI has been previously reported following the first discharge.^54,55^ Nonetheless, the lithium silicate-coated cathode shows lower interfacial impedance.

#### 3.2.1 Lithium Isotope Exchange Experiments on LMR-NMC

**Figure 7:**
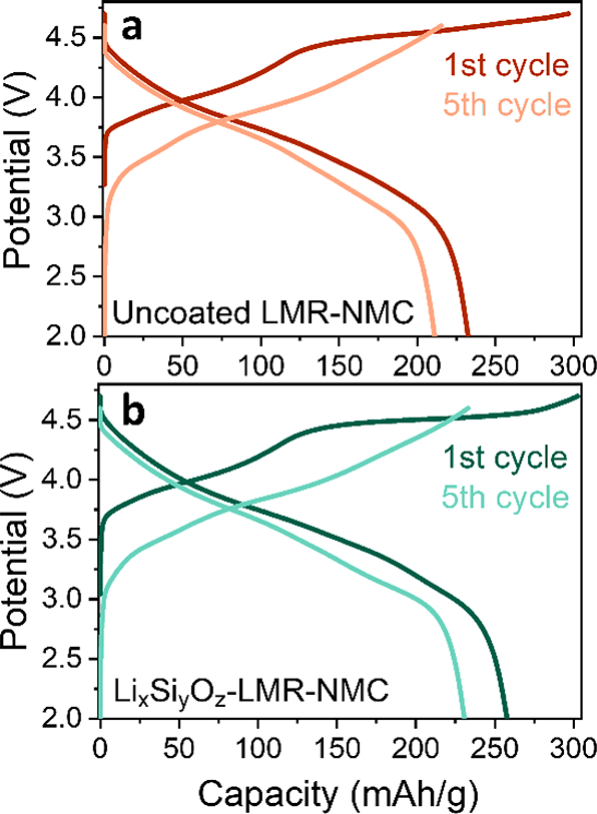
Voltage vs capacity plots for (a) the uncoated LMR-NMC and (b) Li_*x*_Si_*y*_O_*z*_-LMR-NMC cycled vs ^6^Li metal. Representative profiles are shown for the 1st cycle and 5th cycle.

**Figure 8:**
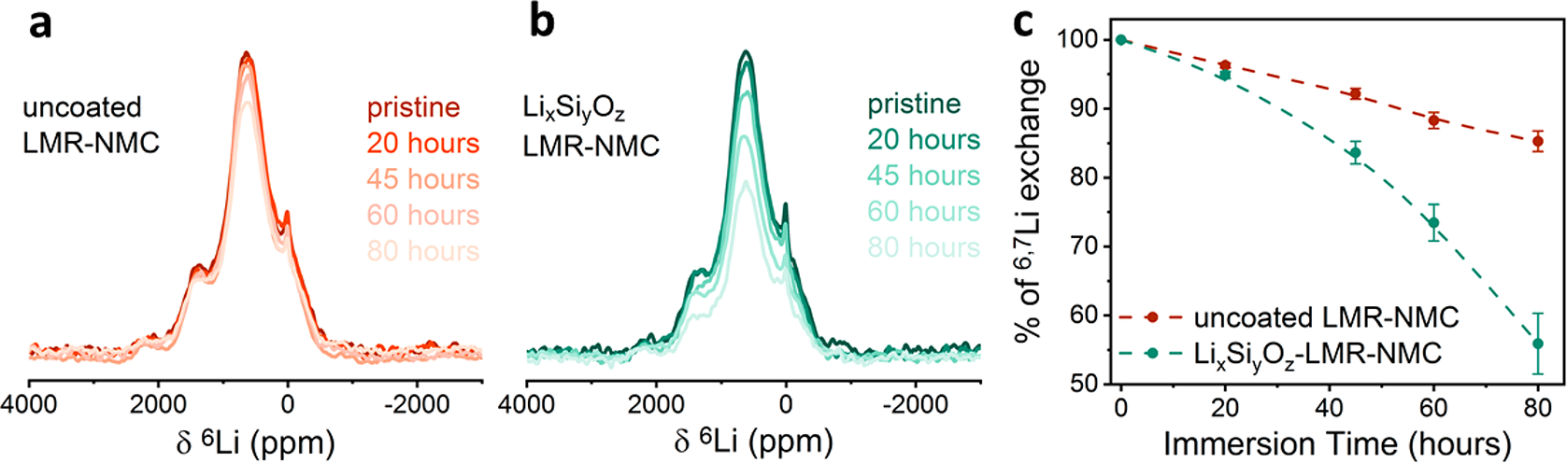
Room temperature magic-angle spinning ^6^Li Hahn echo spectra of (a) uncoated LMR-NMC and (b) Li_*x*_Si_*y*_O_*z*_-LMR-NMC after different immersion times in LP30 (natural isotopes abundance). A relaxation delay of 0.25 s and 4096–25600 scans were used. Spectra were normalized by number of scans, weight of sample, and receiver gain. (c) Percent of exchange for the uncoated (dark red dashed line) and coated (green dashed line) ^6^LMR-NMC vs immersion time in LP30. Experiments were performed at room temperature with a spinning speed of 50 kHz.

Isotope exchange experiments were performed to gain direct insight into ion exchange processes across the CEI. Here we made use of the possibility to detect the two NMR-active lithium isotopes: ^6^Li (7.6% natural abundance) and ^7^Li (92.4% abundance). By following the changes in the amount of one of the isotopes, when two lithium-containing phases varying in their isotope ratios are in contact, we can get insight into ionic mobility. A similar approach has been used for quantification of spontaneous diffusion in the electrode bulk^56,57^ and across the electrode–electrolyte interface.^58,59^ For isotope enrichment, the uncoated LMR-NMC and lithium silicate-coated LMR-NMC were first cycled vs ^6^Li metal for five cycles, with voltage range of 2.0–4.7 V for the first cycle with a C-rate of C/15 and consecutive cycles with the voltage range 2.0–4.6 V and a C-rate of C/10 (representative electrochemistry profiles shown in Figure 7 and Figure S7). The cathodes were then extracted from the cell, rinsed thoroughly with DMC, and immersed in LP30 (natural isotopes abundance). The ^6^Li content in the cathodes, following different immersion times, was determined by ^6^Li magic-angle spinning NMR. Figures 8a and 8b present the ^6^Li spectra of the uncoated and coated LMR-NMC samples at various immersion times. The percentage of isotope exchange was calculated with respect to the initial state (no immersion) and is plotted in Figure 8c (integrated intensity, normalized by sample weight and number of scans, as a function of time is shown in Figure S8). The initial amount of ^6^Li in the uncoated cathode and the Li_*x*_Si_*y*_O_*z*_-coated cathode was similar, yet the slope was significantly different. In the uncoated LMR-NMC a gradual decline in ^6^Li content was observed, reaching 10% decrease after 80 h immersion. A much more pronounced decay was observed for the coated cathode, reaching 55% of its initial ^6^Li content at 80 h.

These results provide direct evidence for improved ion exchange across the artificial CEI and through the bulk of the cathode achieved due to the coating. Because the LMR-NMC particles are 5–10 μm in size,^36^ it is unlikely that they differ in their bulk properties due to the surface treatment. Thus, we suspect the difference in ion exchange is due to increased interfacial transport achieved by coating the particles. Such improvements can be due to suppression of degradation processes, such as prevention of cracks or CEI formation and/or chemical and structural rearrangement. The CEI formed on the uncoated LMR-NMC cathode may be the reason for the inferior Li ion dynamics.

#### 3.2.2 Lithium Isotope Exchange Experiments on Coated TiO_2_

**Figure 9:**
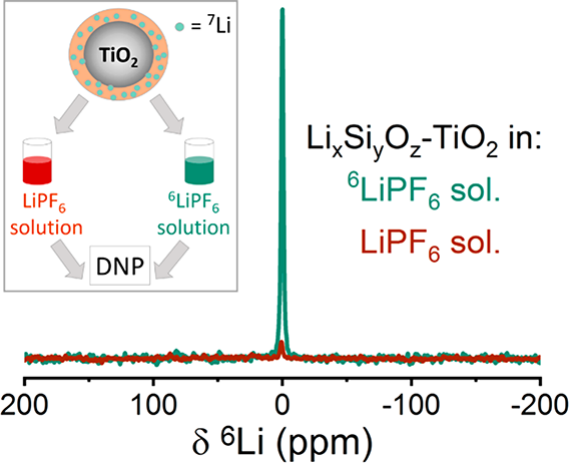
^1^H–^6^Li cross-polarization spectra acquired with exogenous DNP from Li_*x*_Si_*y*_O_*z*_–TiO_2_ after immersion in 0.025 M ^6^LiPF_6_ solution (green line) and 0.025 M LiPF_6_ solution (dark red line). A polarization time of 15 s, contact time of 3.6 ms, and 32 scans were used. Spectra were acquired with microwave irradiation at 100 K, and samples were spun at 10 kHz.

To determine whether the Li_*x*_Si_*y*_O_*z*_ surface layer plays an active role in the improved rate performance, beyond acting as a physical barrier, we performed additional isotope exchange experiments on the coated TiO_2_ particles. Li_*x*_Si_*y*_O_*z*_–TiO_2_ samples were immersed in 0.025 M ^6^LiPF_6_ solution and in a control solution of 0.025 M LiPF_6_ (at natural abundance) and examined with exogenous DNP. The tetrachloroethane ^1^H echo spectra (Figure S9a,b) acquired with and without microwave irradiation showed high polarization and high enhancement factors for both samples. Cross-polarization of this solvent enhanced signal enabled detection of the ^6^Li environments in the coating (Figure 9). The ^6^Li signals were normalized by the ^1^H polarization enhancement factor for each sample to remove any differences due to sample preparation which may lead to different enhancements (after taking into account the weight of the sample, number of scans, and receiver gain). Results from direct polarization of ^6^Li in the coating are shown in Figure S10. In both direct and indirect polarization experiments, the ^6^Li resonance from the coating was significantly larger after immersion in ^6^LiPF_6_ solution compared to the control experiment. This increase is a result of ^6,7^Li exchange between the lithium ions in the enriched electrolyte solution and in the lithium silicate surface layer on the TiO_2_ sample. ^29^Si spectra (Figure S11), acquired with indirect polarization and CPMG detection, were identical with spectra acquired for samples with no immersion, confirming that the coating layer stayed intact following the immersion in LiPF_6_ solution.

Thus, we conclude that lithium sites in the Li_*x*_Si_*y*_O_*z*_ surface layer are exchangeable, strongly suggesting that the coating layer takes part in the transport process between the electrolyte and the cathode. This functionality of the coating layer leads to efficient lithium transport across the CEI, which along with the mechanical and chemical stability it provides to the cathode^36^ results in reduced interfacial resistance and enhanced capacity and rate performance.

## 4 Conclusions

In this work, we demonstrated how ssNMR with increased sensitivity from DNP can be used as an excellent probe for thin protection layers used as artificial CEIs of high-energy cathodes. The remarkable sensitivity gained from exogenous DNP enabled multinuclear characterization of the chemical environments formed with a new MLD coating process based on the alkylated silyllithium precursor. REDOR experiments, possible through DNP, revealed separation between lithium and silicon environments. Endogenous DNP was employed for the first time extending the polarization from bulk to surface. Importantly, the combination of these two DNP approaches, polarizing the outer surface layers with exogenous DNP and inner layers with endogenous DNP, proved to be a powerful structural tool. Insight into the three-dimensional architecture of the surface layers suggests that lithium is distributed across the coating layer in a stacked structure, with monoalkylated silica and SiO_*x*_ groups on the interface of the coating with the electrode and organic moieties facing the interface with the electrolyte.

Furthermore, ssNMR proved to be a valuable tool for directly following ionic mobility, a key parameter for the assessment of the functionality of electrode–electrolyte interfaces. Lithium isotope exchange experiments revealed the enhanced ion transport properties of the coated LMR-NMC samples. Additionally, with sensitivity gained from exogenous DNP we were able to show that the lithium sites in the lithium silicate surface layer are exchangeable, providing direct evidence for the role of the coating in the ion transport process. These results provide atomic-scale rationalization of the EIS measurements and the enhanced rate performance observed for coated cathodes, further establishing the coating’s functionality as an efficient protective surface layer for high-energy cathodes.

We expect the presented ssNMR-DNP methodology will be beneficial in the study of other thin, disordered, and heterogeneous surface layers: for rationally designing artificial CEIs and SEIs as well as for understanding the structure and function of electrochemically and chemically formed interphases in battery cells. The ability to correlate the chemical composition, structure, and transport properties of interfaces and interphases is an essential step for developing high-energy, long-lasting energy storage systems. Thus, the presented methodology forms a promising addition to the characterization toolbox of energy storage materials.

## Supporting Information Available

Additional NMR and DNP experiments and information: room temperature MAS NMR measurements, experimental details of NMR and DNP experiments, representation of DNP approaches, sample preparation for DNP experiment; crystallographic structure of lithium metasilicate and numerical simulations of ^29^Si-{^7^Li} REDOR curves; powder XRD of synthesized Fe-doped TiO_2_, electrochemical cycling data for LMR-NMC and coated LMR-NMC vs ^6^Li and quantification of NMR signal intensity vs immersion time for lithium isotope exchange experiments; electrochemical cycling data for batteries tested with EIS (PDF)

## References

[ref1] Winter M. The Solid Electrolyte Interphase – The Most Important and the Least Understood Solid Electrolyte in Rechargeable Li Batteries. Z. Phys. Chem. 2009, 223 (10–11), 1395–1406. 10.1524/zpch.2009.6086.

[ref2] Xu K. Electrolytes and Interphases in Li-Ion Batteries and Beyond. Chem. Rev. 2014, 114 (23), 11503–11618. 10.1021/cr500003w. 25351820

[ref3] Peled E. The Electrochemical Behavior of Alkali and Alkaline Earth Metals in Nonaqueous Battery Systems—The Solid Electrolyte Interphase Model. J. Electrochem. Soc. 1979, 126 (12), 2047 10.1149/1.2128859.

[ref4] Peled E.; Menkin S. Review—SEI: Past, Present and Future. J. Electrochem. Soc. 2017, 164 (7), A1703–A1719. 10.1149/2.1441707jes.

[ref5] Gauthier M.; Carney T. J.; Grimaud A.; Giordano L.; Pour N.; Chang H.-H.; Fenning D. P.; Lux S. F.; Paschos O.; Bauer C.; Maglia F.; Lupart S.; Lamp P.; Shao-Horn Y. Electrode–Electrolyte Interface in Li-Ion Batteries: Current Understanding and New Insights. J. Phys. Chem. Lett. 2015, 6 (22), 4653–4672. 10.1021/acs.jpclett.5b01727. 26510477

[ref6] Li W.; Song B.; Manthiram A. High-Voltage Positive Electrode Materials for Lithium-Ion Batteries. Chem. Soc. Rev. 2017, 46 (10), 3006–3059. 10.1039/C6CS00875E. 28440379

[ref7] Zeng X.; Zhan C.; Lu J.; Amine K. Stabilization of a High-Capacity and High-Power Nickel-Based Cathode for Li-Ion Batteries. Chem. 2018, 4 (4), 690–704. 10.1016/j.chempr.2017.12.027.

[ref8] Zhao Y.; Zheng K.; Sun X. Addressing Interfacial Issues in Liquid-Based and Solid-State Batteries by Atomic and Molecular Layer Deposition. Joule 2018, 2 (12), 2583–2604. 10.1016/j.joule.2018.11.012.

[ref9] Andre D.; Meiler M.; Steiner K.; Wimmer C.; Soczka-Guth T.; Sauer D. U. Characterization of High-Power Lithium-Ion Batteries by Electrochemical Impedance Spectroscopy. I. Experimental Investigation. J. Power Sources 2011, 196 (12), 5334–5341. 10.1016/j.jpowsour.2010.12.102.

[ref10] Tatara R.; Karayaylali P.; Yu Y.; Zhang Y.; Giordano L.; Maglia F.; Jung R.; Schmidt J. P.; Lund I.; Shao-Horn Y. The Effect of Electrode-Electrolyte Interface on the Electrochemical Impedance Spectra for Positive Electrode in Li-Ion Battery. J. Electrochem. Soc. 2019, 166 (3), A5090–A5098. 10.1149/2.0121903jes.

[ref11] Ohashi T.; Okazaki K.; Fukunaga T.; Ogumi Z.; Abe T. Lithium-Ion Transfer at Cathode-Electrolyte Interface in Diluted Electrolytes Using Electrochemical Impedance Spectroscopy. ChemElectroChem 2020, 7 (7), 1644–1651. 10.1002/celc.202000173.

[ref12] Cheng J.; Sivonxay E.; Persson K. A. Evaluation of Amorphous Oxide Coatings for High-Voltage Li-Ion Battery Applications Using a First-Principles Framework. ACS Appl. Mater. Interfaces 2020, 12 (31), 35748–35756. 10.1021/acsami.0c10000. 32657117

[ref13] Wang C.; Aoyagi K.; Wisesa P.; Mueller T. Lithium Ion Conduction in Cathode Coating Materials from On-the-Fly Machine Learning. Chem. Mater. 2020, 32 (9), 3741–3752. 10.1021/acs.chemmater.9b04663.

[ref14] Xu S.; Jacobs R. M.; Nguyen H. M.; Hao S.; Mahanthappa M.; Wolverton C.; Morgan D. Lithium Transport through Lithium-Ion Battery Cathode Coatings. J. Mater. Chem. A 2015, 3 (33), 17248–17272. 10.1039/C5TA01664A.

[ref15] Shi S.; Qi Y.; Li H.; Hector L. G. Defect Thermodynamics and Diffusion Mechanisms in Li2CO 3 and Implications for the Solid Electrolyte Interphase in Li-Ion Batteries. J. Phys. Chem. C 2013, 117 (17), 8579–8593. 10.1021/jp310591u.

[ref16] Pecher O.; Carretero-González J.; Griffith K. J.; Grey C. P. Materials’ Methods: NMR in Battery Research. Chem. Mater. 2017, 29 (1), 213–242. 10.1021/acs.chemmater.6b03183.

[ref17] Haber S.; Leskes M. What Can We Learn from Solid State NMR on the Electrode – Electrolyte Interface ?. Adv. Mater. 2018, 30 (41), 1706496 10.1002/adma.201706496. 29889328

[ref18] Heitjans P.; Wilkening M. Ion Dynamics at Interfaces: Nuclear Magnetic Resonance Studies. MRS Bull. 2009, 34, 915–922. 10.1557/mrs2009.213.

[ref19] Chandran C. V.; Heitjans P. Solid-State NMR Studies of Lithium Ion Dynamics Across Materials Classes, 1st ed.; Elsevier Ltd.: 2016.

[ref20] Rossini A. J.; Zagdoun A.; Lelli M.; Lesage A.; Copéret C.; Emsley L. Dynamic Nuclear Polarization Surface Enhanced NMR Spectroscopy. Acc. Chem. Res. 2013, 46 (9), 1942–1951. 10.1021/ar300322x. 23517009

[ref21] Maly T.; Debelouchina G. T.; Bajaj V. S.; Hu K.-N.; Joo C.-G.; Mak-Jurkauskas M. L.; Sirigiri J. R.; van der Wel P. C. A.; Herzfeld J.; Temkin R. J.; et al. Dynamic Nuclear Polarization at High Magnetic Fields. J. Chem. Phys. 2008, 128 (5), 052211 10.1063/1.2833582. 18266416 PMC2770872

[ref22] Lilly Thankamony A. S.; Wittmann J. J.; Kaushik M.; Corzilius B. Dynamic Nuclear Polarization for Sensitivity Enhancement in Modern Solid-State NMR. Prog. Nucl. Magn. Reson. Spectrosc. 2017, 102–103, 120–195. 10.1016/j.pnmrs.2017.06.002. 29157490

[ref23] Rankin A. G. M.; Trébosc J.; Pourpoint F.; Amoureux J. P.; Lafon O. Recent Developments in MAS DNP-NMR of Materials. Solid State Nucl. Magn. Reson. 2019, 101, 116–143. 10.1016/j.ssnmr.2019.05.009. 31189121

[ref24] Leskes M.; Kim G.; Liu T.; Michan A. L.; Aussenac F.; Dorffer P.; Paul S.; Grey C. P. Surface-Sensitive NMR Detection of the Solid Electrolyte Interphase Layer on Reduced Graphene Oxide. J. Phys. Chem. Lett. 2017, 8 (5), 1078–1085. 10.1021/acs.jpclett.6b02590. 28195488

[ref25] Hestenes J. C.; Ells A. W.; Navarro Goldaraz M.; Sergeyev I. V.; Itin B.; Marbella L. E. Reversible Deposition and Stripping of the Cathode Electrolyte Interphase on Li_2_RuO_3_. Front. Chem. 2020, 8 (Aug), 1–9. 32850679 10.3389/fchem.2020.00681 PMC7417863

[ref26] Jin Y.; Kneusels N. J. H.; Marbella L. E.; Castillo-Martínez E.; Magusin P. C. M. M.; Weatherup R. S.; Jónsson E.; Liu T.; Paul S.; Grey C. P. Understanding Fluoroethylene Carbonate and Vinylene Carbonate Based Electrolytes for Si Anodes in Lithium Ion Batteries with NMR Spectroscopy. J. Am. Chem. Soc. 2018, 140 (31), 9854–9867. 10.1021/jacs.8b03408. 29979869

[ref27] Corzilius B.; Michaelis V. K.; Penzel S. A.; Ravera E.; Smith A. A.; Luchinat C.; Griffin R. G. Dynamic Nuclear Polarization of 1 H, 13 C, and 59 Co in a Tris(Ethylenediamine)Cobalt(III) Crystalline Lattice Doped with Cr(III). J. Am. Chem. Soc. 2014, 136 (33), 11716–11727. 10.1021/ja5044374. 25069794 PMC4140501

[ref28] Jacquinot J. F.; Wenckebach W. T.; Goldman M.; Abragam A. Polarization and NMR Observation of Ca43 Nuclei in CaF2. Phys. Rev. Lett. 1974, 32 (20), 1096–1097. 10.1103/PhysRevLett.32.1096.

[ref29] Brun E.; Derighetti B.; Hundt E. E.; Niebuhr H. H. NMR of 17O in Ruby with Dynamic Polarization Techniques. Phys. Lett. A 1970, 31 (8), 416–417. 10.1016/0375-9601(70)90371-3.

[ref30] Derighetti B.; Hafner S.; Marxer H.; Rager H. NMR of 29Si and 25Mg In Mg2SiO4 with Dynamic Polarization Technique. Phys. Lett. A 1978, 66 (2), 150–152. 10.1016/0375-9601(78)90023-3.

[ref31] Kaushik M.; Bahrenberg T.; Can T. V.; Caporini M. A.; Silvers R.; Heiliger J.; Smith A. A.; Schwalbe H.; Griffin R. G.; Corzilius B. Gd(III) and Mn(II) Complexes for Dynamic Nuclear Polarization: Small Molecular Chelate Polarizing Agents and Applications with Site-Directed Spin Labeling of Proteins. Phys. Chem. Chem. Phys. 2016, 18, 27205–27218. 10.1039/C6CP04623A. 27545112 PMC5053914

[ref32] Chakrabarty T.; Goldin N.; Feintuch A.; Houben L.; Leskes M. Paramagnetic Metal-Ion Dopants as Polarization Agents for Dynamic Nuclear Polarization NMR Spectroscopy in Inorganic Solids. ChemPhysChem 2018, 19 (17), 2139–2142. 10.1002/cphc.201800462. 29770999

[ref33] Jardón-Álvarez D.; Reuveni G.; Harchol A.; Leskes M. Enabling Natural Abundance 17O Solid-State NMR by Direct Polarization from Paramagnetic Metal Ions. J. Phys. Chem. Lett. 2020, 11 (14), 5439–5445. 10.1021/acs.jpclett.0c01527. 32551646 PMC7370305

[ref34] Wolf T.; Kumar S.; Singh H.; Chakrabarty T.; Aussenac F.; Frenkel A. I.; Major D. T.; Leskes M. Endogenous Dynamic Nuclear Polarization for Natural Abundance 17 O and Lithium NMR in the Bulk of Inorganic Solids. J. Am. Chem. Soc. 2019, 141 (1), 451–462. 10.1021/jacs.8b11015. 30525555

[ref35] Harchol A.; Reuveni G.; Ri V.; Thomas B.; Carmieli R.; Herber R. H.; Kim C.; Leskes M. Endogenous Dynamic Nuclear Polarization for Sensitivity Enhancement in Solid-State NMR of Electrode Materials. J. Phys. Chem. C 2020, 124 (ii), 7082–7090. 10.1021/acs.jpcc.0c00858. PMC7133110 32273937

[ref36] Rosy; Haber S.; Evenstein E.; Saha A.; Brontvein O.; Kratish Y.; Bravo-Zhivotovskii D.; Apeloig Y.; Leskes M.; Noked M. Alkylated LixSiyOz Coating for Stabilization of Li-Rich Layered Oxide Cathodes. Energy Storage Mater. 2020, 33, 268–275. 10.1016/j.ensm.2020.08.015.

[ref37] Jiang X.; Herricks T.; Xia Y. Monodispersed Spherical Colloids of Titania: Synthesis, Characterization, and Crystallization. Adv. Mater. 2003, 15 (14), 1205–1209. 10.1002/adma.200305105.

[ref38] Ates M. N.; Mukerjee S.; Abraham K. M. A High Rate Li-Rich Layered MNC Cathode Material for Lithium-Ion Batteries. RSC Adv. 2015, 5 (35), 27375–27386. 10.1039/C4RA17235C.

[ref39] Thakur R. S.; Kurur N. D.; Madhu P. Swept-Frequency Two-Pulse Phase Modulation for Heteronuclear Dipolar Decoupling in Solid-State NMR. Chem. Phys. Lett. 2006, 426, 459–463. 10.1016/j.cplett.2006.06.007.

[ref40] Bein T.; Carver R. F.; Farlee R. D.; Stucky G. D. Solid-State 29Si NMR and Infrared Studies of the Reactions of Mono- and Polyfunctional Silanes with Zeolite Y Surfaces. J. Am. Chem. Soc. 1988, 110, 4546–4553. 10.1021/ja00222a010.

[ref41] Mackenzie K. J.; Smith M. Multinuclear Solid-State NMR of Inorganic Materials. In Pergamon Materials Series; Cahn R. W., Ed.; Elsevier Science Ltd.: UK, 2002.

[ref42] Sindorf D. W.; Maciel G. E. 13C CP/MAS NMR Study of Molecular Motion in n-Alkylsilanes Bonded to the Silica Surface. J. Am. Chem. Soc. 1983, 105 (7), 1848–1851. 10.1021/ja00345a028.

[ref43] Michael G. E.; Sindorf D. W.; Bartuska V. J. Characterization of Silica-Attached Systems by 29Si and 13C Cross-Polarization and Magic-Angle Spinning Nuclear Magentic Resonance. J. Chromatogr. 1981, 205 (205), 438–443. 10.1016/S0021-9673(00)82673-4.

[ref44] Dupré N.; Cuisinier M.; Guyomard D. Electrode/Electrolyte Interface Studies in Lithium Batteries Using NMR. Interface 2011, 20 (3), 61–67.

[ref45] Gullion T. Introduction to Rotational-Echo, Double-Resonance NMR. Concepts Magn. Reson. 1998, 10 (5), 277–289. 10.1002/(SICI)1099-0534(1998)10:5<277::AID-CMR1>3.0.CO;2-U.

[ref46] Yamada M.; Inaba A.; Ueda A.; Matsumoto K.; Iwasaki T.; Ohzuku T. Reaction Mechanism of “SiO”-Carbon Composite-Negative Electrode for High-Capacity Lithium-Ion Batteries. J. Electrochem. Soc. 2012, 159 (10), A1630–A1635. 10.1149/2.018210jes.

[ref47] Hirose T.; Morishita M.; Yoshitake H.; Sakai T. Study of Structural Changes That Occurred during Charge/Discharge of Carbon-Coated SiO Anode by Nuclear Magnetic Resonance. Solid State Ionics 2017, 303, 154–160. 10.1016/j.ssi.2017.03.004.

[ref48] Veshtort M.; Griffin R. G. SPINEVOLUTION: A Powerful Tool for the Simulation of Solid and Liquid State NMR Experiments. J. Magn. Reson. 2006, 178 (2), 248–282. 10.1016/j.jmr.2005.07.018. 16338152

[ref49] Akbey Ü.; Altin B.; Linden A.; Özçelik S.; Gradzielski M.; Oschkinat H. Dynamic Nuclear Polarization of Spherical Nanoparticles. Phys. Chem. Chem. Phys. 2013, 15 (47), 20706–20716. 10.1039/c3cp53095g. 24192797

[ref50] Stoll S.; Schweiger A. EasySpin, a Comprehensive Software Package for Spectral Simulation and Analysis in EPR. J. Magn. Reson. 2006, 178 (1), 42–55. 10.1016/j.jmr.2005.08.013. 16188474

[ref51] Sofia A.; Thankamony L.; Wittmann J. J.; Kaushik M.; Corzilius B. Dynamic Nuclear Polarization for Sensitivity Enhancement in Modern Solid-State NMR. Prog. Nucl. Magn. Reson. Spectrosc. 2017, 102–103, 120–195. 10.1016/j.pnmrs.2017.06.002. 29157490

[ref52] Xie H.; Du K.; Hu G.; Duan J.; Peng Z.; Zhang Z.; Cao Y. Synthesis of LiNi0.8Co0.15Al0.05O2 with 5-Sulfosalicylic Acid as a Chelating Agent and Its Electrochemical Properties. J. Mater. Chem. A 2015, 3 (40), 20236–20243. 10.1039/C5TA05266A.

[ref53] Schipper F.; Dixit M.; Kovacheva D.; Talianker M.; Haik O.; Grinblat J.; Erickson E. M.; Ghanty C.; Major D. T.; Markovsky B.; et al. Stabilizing Nickel-Rich Layered Cathode Materials by a High-Charge Cation Doping Strategy: Zirconium-Doped LiNi0.6Co0.2Mn0.2O2. J. Mater. Chem. A 2016, 4 (41), 16073–16084. 10.1039/C6TA06740A.

[ref54] Li Q.; Wang Y.; Wang X.; Sun X.; Zhang J. N.; Yu X.; Li H. Investigations on the Fundamental Process of Cathode Electrolyte Interphase Formation and Evolution of High-Voltage Cathodes. ACS Appl. Mater. Interfaces 2020, 12 (2), 2319–2326. 10.1021/acsami.9b16727. 31872999

[ref55] Liu H.; Harris K. J.; Jiang M.; Wu Y.; Goward G. R.; Botton G. A. Unraveling the Rapid Performance Decay of Layered High-Energy Cathodes: From Nanoscale Degradation to Drastic Bulk Evolution. ACS Nano 2018, 12 (3), 2708–2718. 10.1021/acsnano.7b08945. 29505239

[ref56] Liu H.; Choe M.; Enrique R. A.; Orvañanos B.; Zhou L.; Liu T.; Thornton K.; Grey C. P. Effects of Antisite Defects on Li Diffusion in LiFePO 4 Revealed by Li Isotope Exchange. J. Phys. Chem. C 2017, 121 (22), 12025–12036. 10.1021/acs.jpcc.7b02819.

[ref57] Murakami M.; Shimizu S.; Noda Y.; Takegoshi K.; Arai H.; Uchimoto Y.; Ogumi Z. Spontaneous Lithium Transportation via LiMn2O4/Electrolyte Interface Studied by 6/7Li Solid-State Nuclear Magnetic Resonance. Electrochim. Acta 2014, 147, 540–544. 10.1016/j.electacta.2014.09.155.

[ref58] Ilott A. J.; Jerschow A. Probing Solid-Electrolyte Interphase (SEI) Growth and Ion Permeability at Undriven Electrolyte–Metal Interfaces Using 7Li NMR. J. Phys. Chem. C 2018, 122, 12598–12604. 10.1021/acs.jpcc.8b01958.

[ref59] Gunnarsdóttir A. B.; Vema S.; Menkin S.; Marbella L. E.; Grey C. P. Investigating the Effect of a Fluoroethylene Carbonate Additive on Lithium Deposition and the Solid Electrolyte Interphase in Lithium Metal Batteries Using: In Situ NMR Spectroscopy. J. Mater. Chem. A 2020, 8 (30), 14975–14992. 10.1039/D0TA05652A.

