# Supplementary material for: Structure and Functionality of an Alkylated LixSiyOz Interphase for High-Energy Cathodes from DNP-ssNMR Spectroscopy
Source: J Am Chem Soc. 2021 Mar 22;143(12):4694–704. doi: 10.1021/jacs.1c00215 (PMC8017524; doi:10.1021/jacs.1c00215)
Supplement: Supplementary file 1 — ja1c00215_si_001.pdf [file ja1c00215_si_001.pdf]

Supporting Information

Structure and Functionality of an Alkylated $Li_xSi_yO_z$ Interphase for High Energy Cathodes from DNP-ssNMR Spectroscopy

Shira Haber(1), Rosy(2), Arka Saha(3), Olga Brontvein(4), Raanan Carmieli(4), Arava Zohar(1), Malachi Noked(3), and Michal Leskes(1)*

(1) Department of Materials and Interfaces, Weizmann Institute of Science, Rehovot, Israel, 7610001.

(2) Department of Chemistry, Indian Institute of Technology BHU, Varanasi, India 221005

(3) Department of Chemistry, Bar-Ilan University, Ramat Gan, Israel. Bar-Ilan Institute of Nanotechnology and Advanced Materials, Ramat Gan, Israel.

(4) Department of Chemical Research Support, Weizmann Institute of Science, Israel, 7610001.

*michal.leskes@weizmann.ac.il

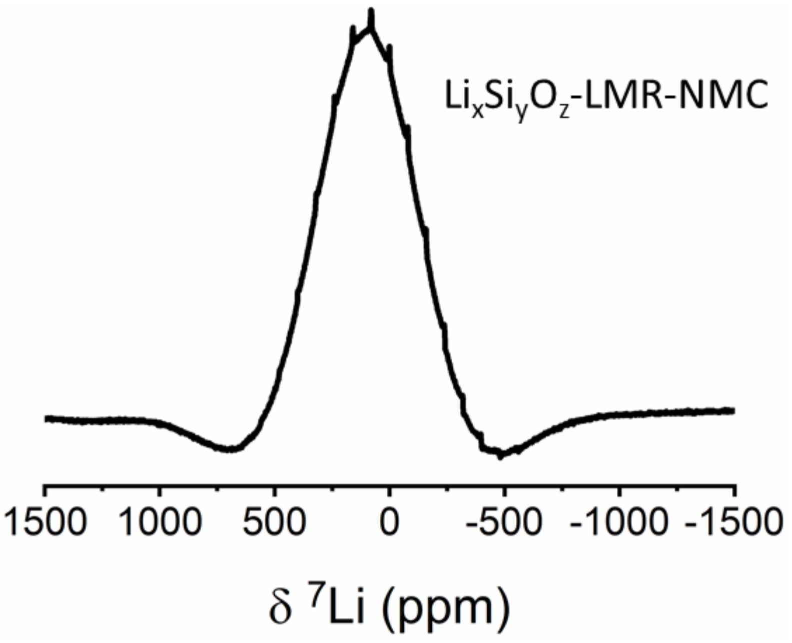

Figure S1

^{7}Li MAS NMR spectrum of $Li_xSi_yO_z$ coated LMR-NMC acquired at room temperature, with a 4 mm probe. Spectrum was acquired with 8192 scans and a recovery delay of 1 s. Spinning speed of 10 kHz was used.

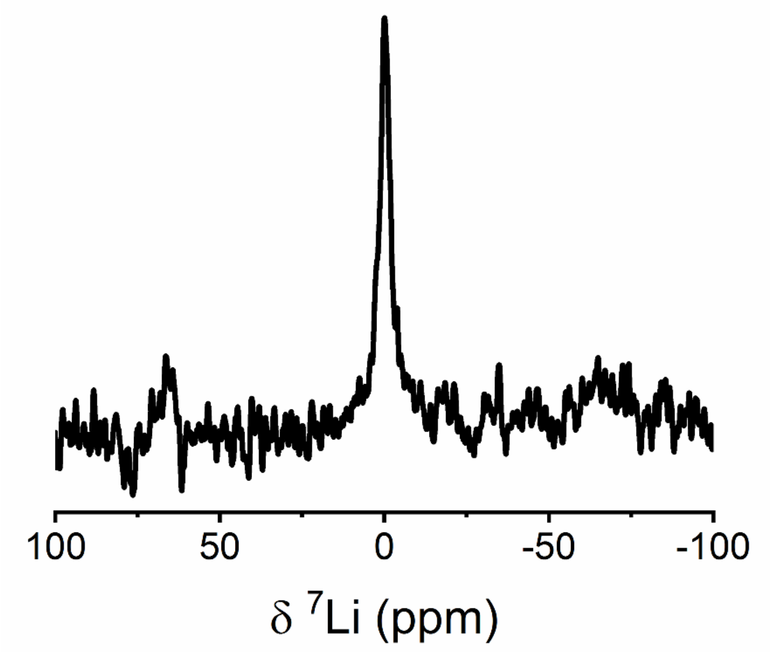

Figure S2. ^{7}Li MAS NMR spectrum of $Li_xSi_yO_z$ coated TiO_2 acquired at room temperature, with a 4 mm probe, enabling the use of 100 mg of material. Spectrum was acquired with 2224 scans and a recovery delay of 40 s with an overall experiment time of about 25 hours. Spinning speed of 10 kHz was used.

Table S1. Sample preparation for Exogeneous DNP experiments

Figure	Weight [mg]	Radical Solution added
1(a)	22.0	25 µl of 16mM TEKPol in TCE
1(b), 4(d)	16	10 µl of 16mM TEKPol in TCE
1(c)	18	8 µl of 16mM TEKPol in TCE*
1(d), 2(b), 4(b)	25.6	15 µl of 16mM TEKPol in TCE
7, S10, S11	20.11 (after immersion in 0.025 M $^{6}LiPF_6$ solution)	10 µl of 16mM TEKPol in TCE*
7, S10, S11, S12	20.36 (after immersion in 0.025 M $LiPF_6$ solution)	10 µl of 16mM TEKPol in TCE*

* powder and nitroxide solution were mixed together in a mortar and then scraped into the rotor.

Table S2. Experimental data for room temperature ssNMR experiments

Sample	Experiment	RF amplitude [kHz]	Relaxation delay [s]	Number of scans	MAS [kHz]
$Li_xSi_yO_z$-TiO_2	^{7}Li direct	125	40	2224	10
$Li_xSi_yO_z$-LMR-NMC	^{6}Li Hahn echo	111	0.25	4096 -25600	50
	^{7}Li Hahn echo	125	1	8192	10

Table S3. Experimental data for Endogenous DNP experiments.

Sample	Experiment	RF amplitude [kHz]	Relaxation delay [s]	Number of scans	MAS [kHz]
$Li_xSi_yO_z$-Fe-TiO_2	^{7}Li direct (field sweep)	68	20	4	10
	^{7}Li direct	68	33	128	10
	^{29}Si CPMG	66	300	126	10

Table S4. Experimental data for Exogenous DNP experiments.

Sample	Experiment	RF amplitude [kHz]	Relaxation delay [s]	Contact time [ms]	Decoupling	Number of scans	MAS [kHz]
$Li_xSi_yO_z$-TiO_2	^{1}H Hahn echo	105	45	N/A	N/A	2	10
	^{1}H-^{29}Si cross polarization	67 (^{29}Si) 60 (^{1}H ramped)	6	2	N/A	3072	10
	^{1}H-^{13}C cross polarization	45 60 (^{1}H ramped)	5	1	swfTPPM with 76.6 kHz	128	10
	^{1}H-^{7}Li cross polarization	61 (^{7}Li) 60 (^{1}H ramped)	10	1	N/A	256	10
	^{29}Si CPMG	66	120	N/A	N/A	192	10
	^{7}Li direct	61	100	N/A	N/A	8	10
	^{1}H-$^{29}Si\{^{7}Li\}$ cross polarization REDOR	105 (^{1}H) 61 (^{7}Li) 67 (^{29}Si) 60 (^{1}H ramped)	5-6	2	N/A	4096-8800	10
Lithium metasilicate	^{7}Li-$^{29}Si\{^{7}Li\}$ cross polarization REDOR	61 (^{7}Li) 66 (^{29}Si) 47 (^{7}Li ramped)	90	4	N/A	48	10
$Li_xSi_yO_z$-TiO_2 Isotope exchange	^{6}Li direct	83	40 and 400	N/A	N/A	8	10
	^{1}H-^{6}Li cross polarization	83 (^{6}Li) 60 (^{1}H ramped)	15-16	3.6	N/A	32	10
	^{1}H-^{29}Si cross polarization -CPMG	66 (^{29}Si) 60 (^{1}H ramped)	3.5	2	N/A	128	10

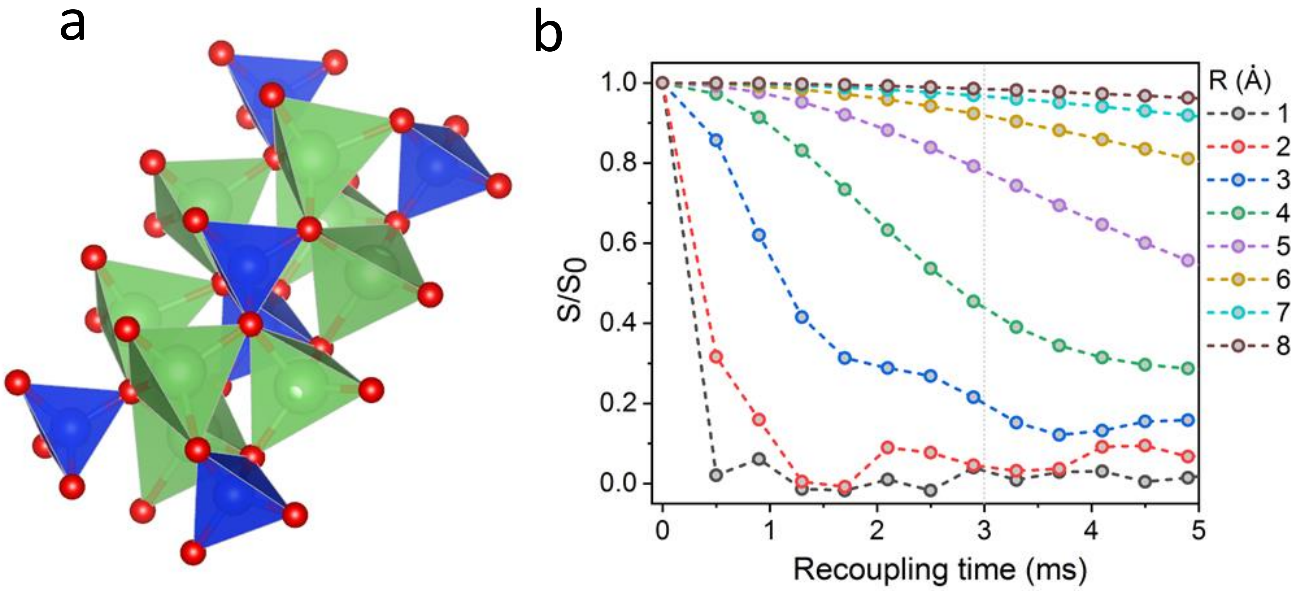

Figure S3. (a) The polyhedral structure of lithium metasilicate showing the proximity of silicon (blue tetrahedra) and lithium (green tetrahedra) linked through oxygen (red). Nearest Si-Li distance is 3Å. (b) Simulation performed with SPINEVOLUTION of $^{29}Si\{^{7}Li\}$ REDOR dephasing of a single spin pair with varying internuclear distance. The ^{7}Li site was simulated with the quadrupolar coupling constant estimated from the DNP experiments on the coated TiO_2 sample, with C_q of 76 kHz and η=0. The dashed line corresponds to the recoupling time used in the experiment. The simulation suggests that for a spin pair with internuclear distance larger than 6Å no significant dephasing can be expected within 3 ms.

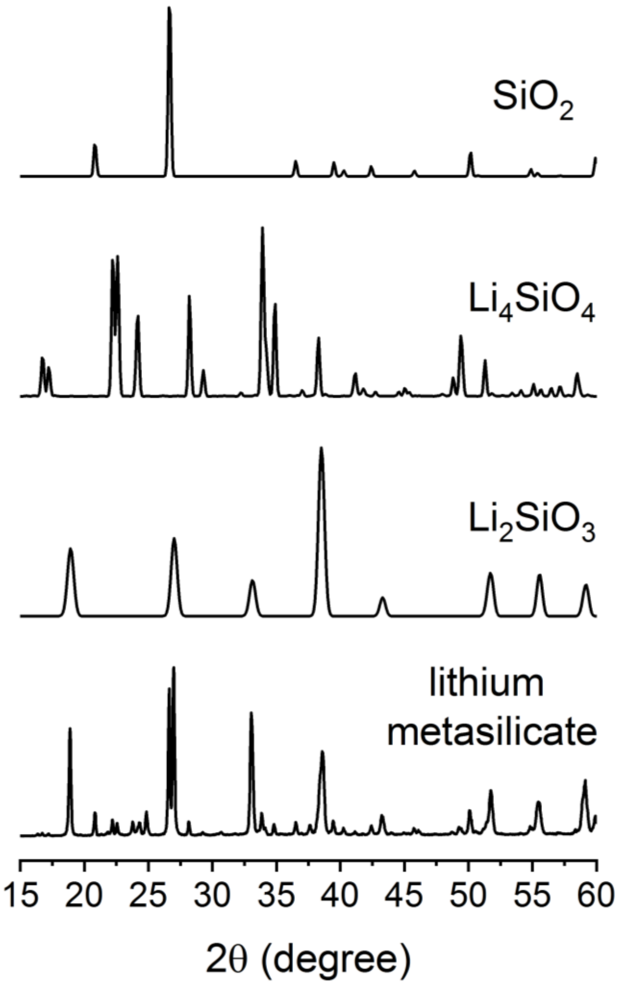

Figure S4. X-ray diffraction of commercial lithium metasilicate, showing additional impurities.[2,3]

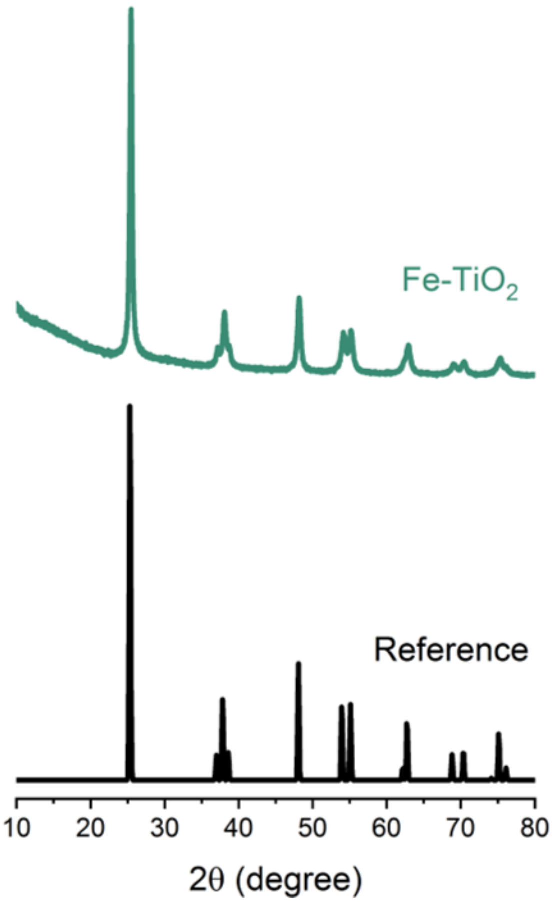

Figure S5. X-ray diffraction of the synthesized iron doped TiO_2 and reference diffraction pattern.[4]

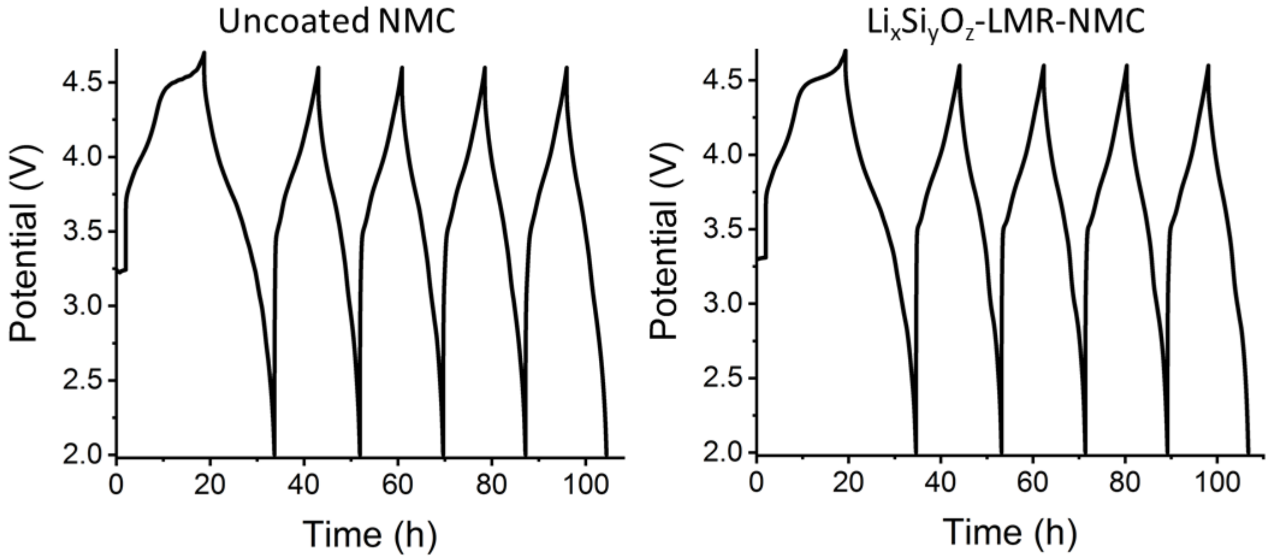

Figure S6. Time vs potential for the uncoated LMR-NMC and $Li_xSi_yO_z$-LMR-NMC vs Li metal. Voltage range for first cycle was 2.0 - 4.7 V with c-rate of C/15 and consecutive cycles were run from 2.0 - 4.6 V with c-rate of C/10.

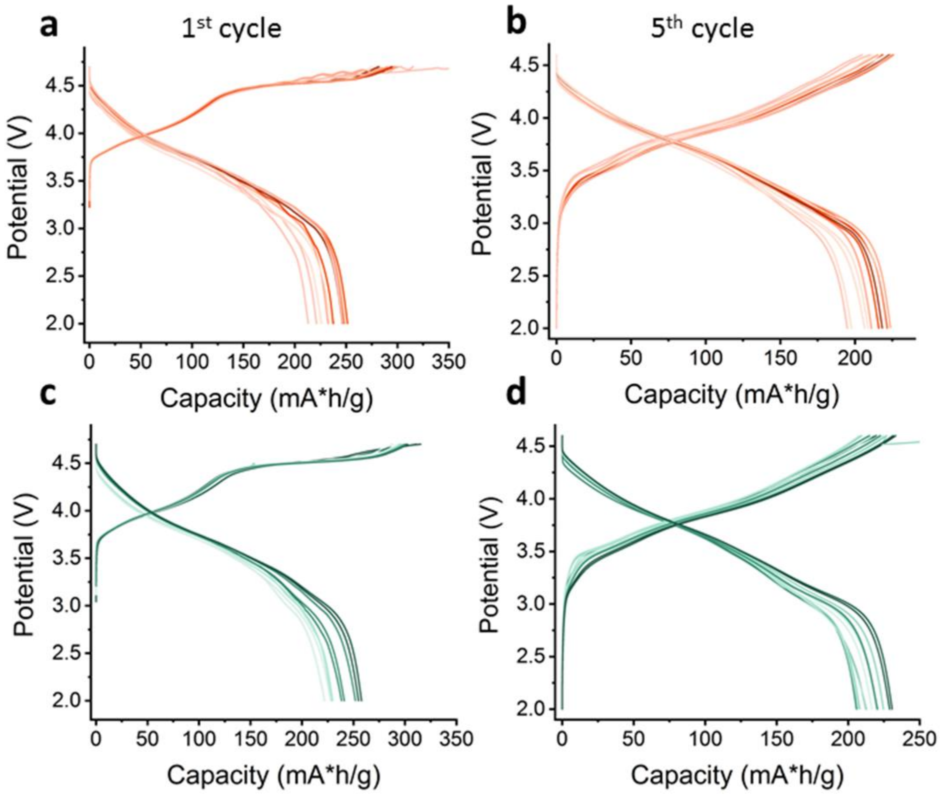

Figure S7. Capacity vs voltage for the uncoated LMR-NMC (a and b) and $Li_xSi_yO_z$-LMR-NMC (c and d) vs ^{6}Li metal, for 1st cycle (left) and 5th cycle (right). Voltage range for first cycle was 2.0 - 4.7 V with c-rate of C/15 and voltage range for fifth cycle was 2.0 - 4.6 V with c-rate of C/10.

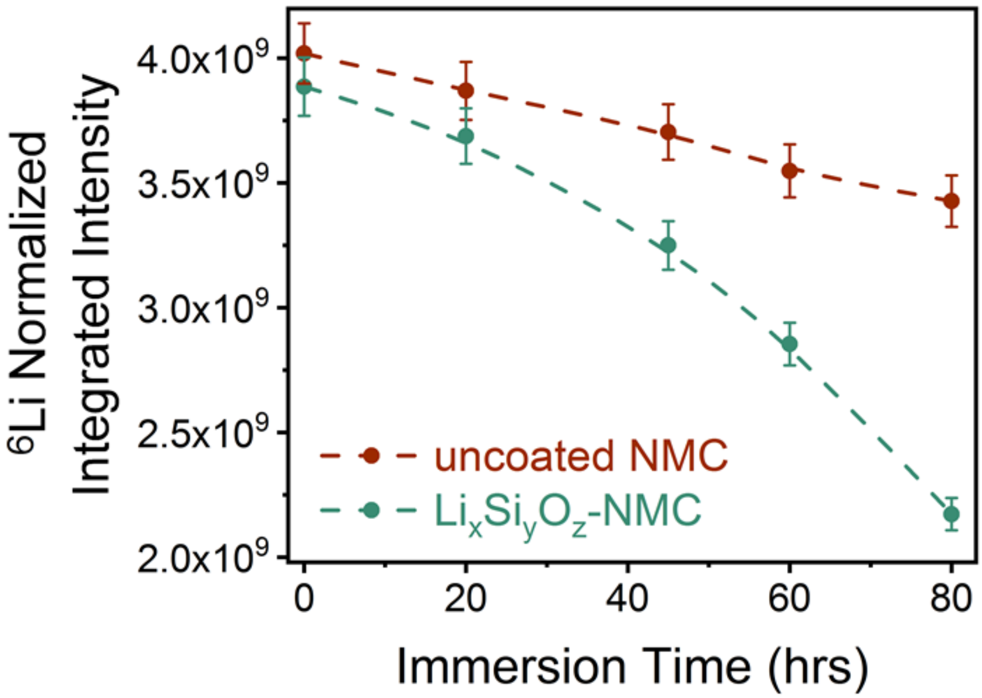

Figure S8. Integrated intensity of ^{6}Li spectra of uncoated (dark red) and lithium-silicate coated LMR-NMC (green) as a function of the immersion time in LP30.

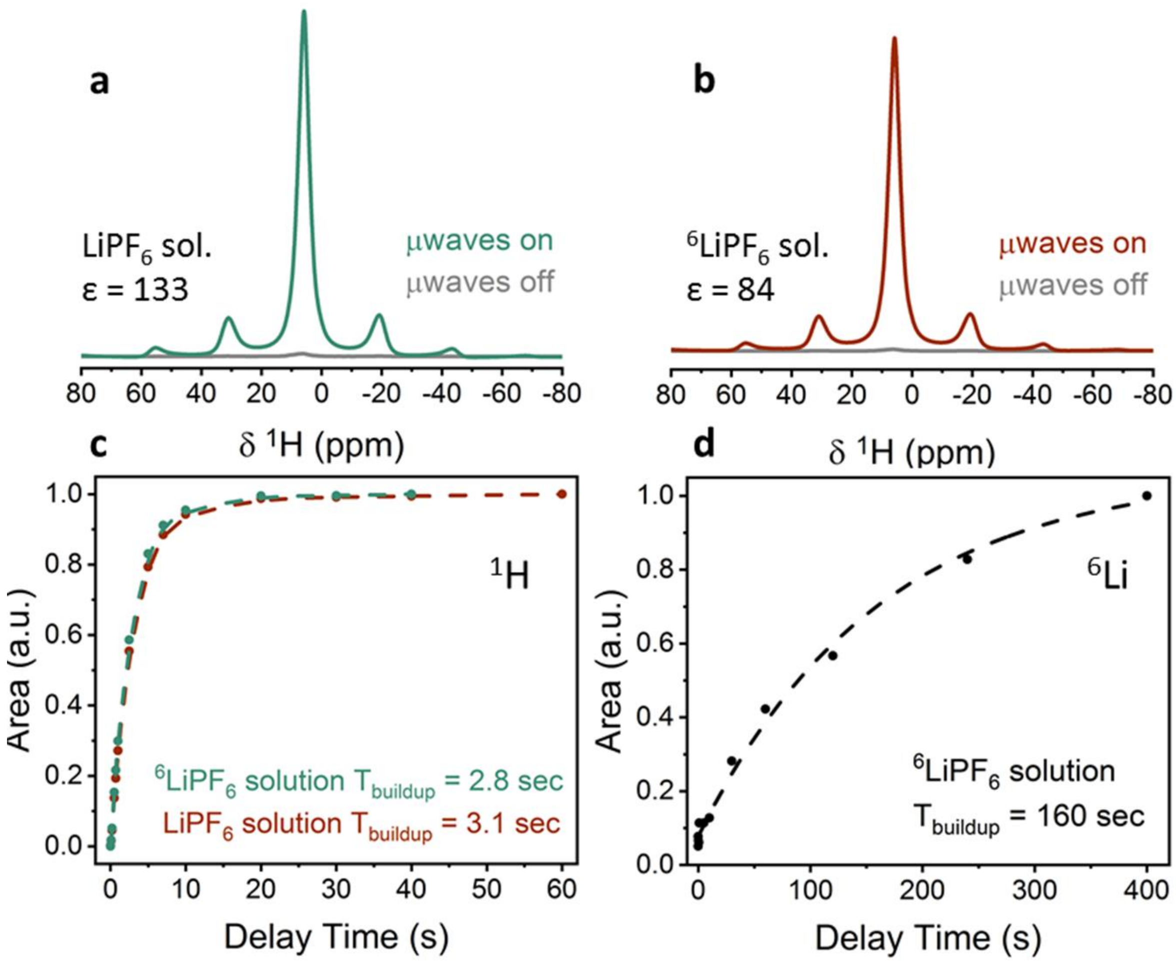

Figure S9. ^{1}H rotor synchronized Hahn echo spectra of coated TiO_2 sample, after immersion in $LiPF_6$ (a) and $^{6}LiPF_6$ (b) solutions, acquired with and without µwave irradiation using a polarization time of 20 s and 2 scans. Spinning sidebands are marked with an asterix. (c) ^{1}H saturation recovery measurements performed on the coated sample after immersion in $^{6}LiPF_6$ (green line) and $LiPF_6$ (red line) solutions, fitted with a mono-exponential function. (d) ^{6}Li saturation recovery measurement of the lithium-silicate coated TiO_2 after immersion in $^{6}LiPF_6$ solution. Samples were spun with 10 kHz spinning speed, at 100 K.

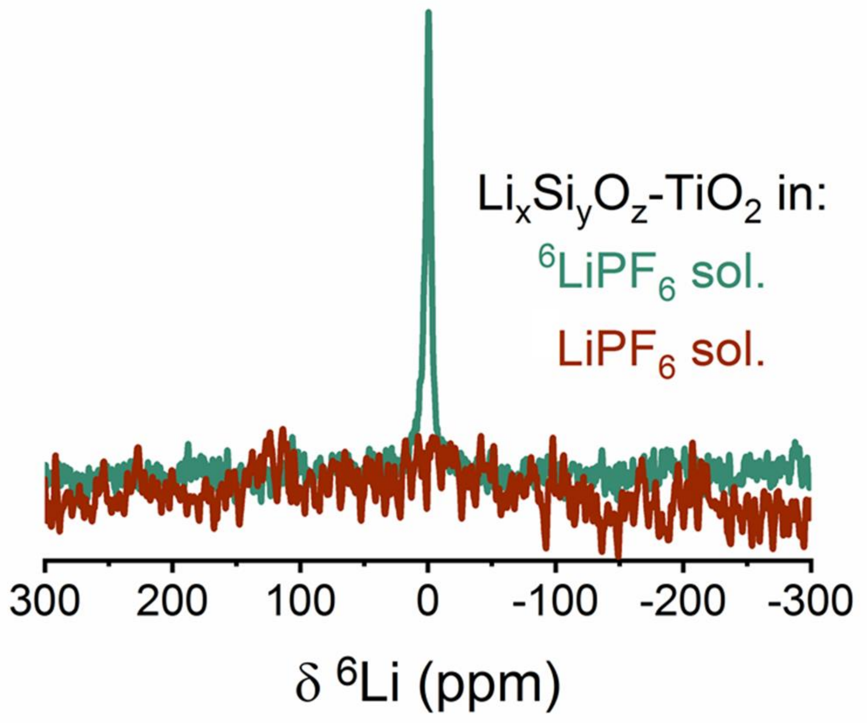

Figure S10. Direct polarization of ^6Li signal of $Li_xSi_yO_z$-TiO_2 after immersion in 0.025 M $^6LiPF_6$ solution (green) and 0.025 M $LiPF_6$ solution (dark red). Polarization time of 40 s (for samples immersed in $^6LiPF_6$ solution) and 400 s (for samples immersed in $LiPF_6$ solution) and 8 scans were used, with spinning speed of 10 kHz and at 100 K.

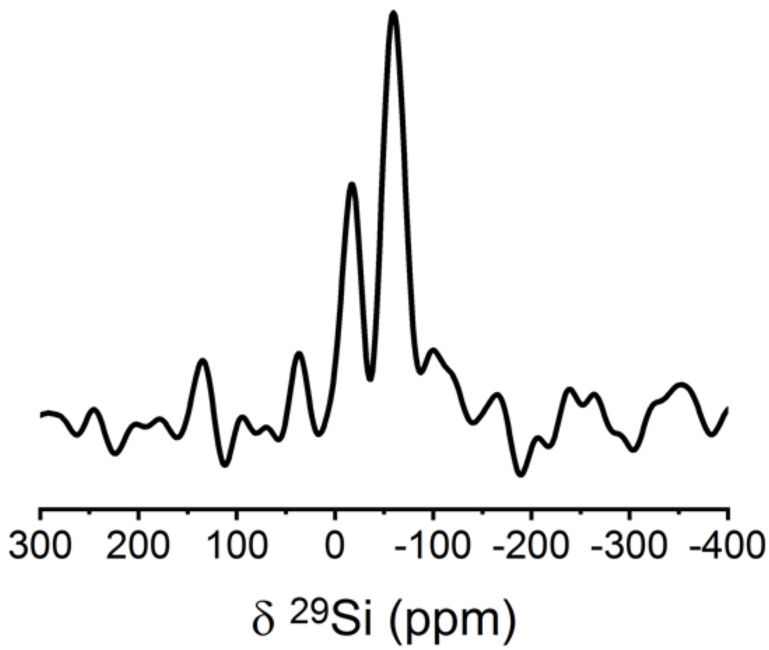

Figure S11. ^{1}H-^{29}Si cross polarization spectrum acquired with µwaves and using CPMG detection for $Li_xSi_yO_z$-TiO_2 after immersion in 0.025 M $LiPF_6$ solution. Relaxation delay of 3.5 s, contact time of 2 ms, and 128 scans were used, with 10 kHz spinning speed, at 100 K.

References:

(1) Thakur, R. S.; Kurur, N. D.; Madhu, P. Swept-Frequency Two-Pulse Phase Modulation for Heteronuclear Dipolar Decoupling in Solid-State NMR. *Chem. Phys. Lett.* **2006**, *426*, 459–463. https://doi.org/10.1016/j.cplett.2006.06.007.

(2) d'Amour, H.; Denner, W.; Schulz, H. Structure Determination of α-Quartz up to 68 x 10 8 Pa . *Acta Crystallogr. Sect. B Struct. Crystallogr. Cryst. Chem.* **1979**, *35* (3), 550–555. https://doi.org/10.1107/s056774087900412x.

(3) Grasso, M. L.; Blanco, M. V.; Cova, F.; González, J. A.; Arneodo Larochette, P.; Gennari, F. C. Evaluation of the Formation and Carbon Dioxide Capture by Li4SiO4 Using: In Situ Synchrotron Powder X-Ray Diffraction Studies. *Phys. Chem. Chem. Phys.* **2018**, *20* (41), 26570–26579. https://doi.org/10.1039/c8cp03611j.

(4) Leinekugel-le-Cocq-Errien, A. Y.; Deniard, P.; Jobic, S.; Gautier, E.; Evain, M.; Aubin, V.; Bart, F. Structural Characterization of the Hollandite Host Lattice for the Confinement of Radioactive Cesium: Quantification of the Amorphous Phase Taking into Account the Incommensurate Modulated Character of the Crystallized Part. *J. Solid State Chem.* **2007**, *180* (1), 322–330. https://doi.org/10.1016/j.jssc.2006.10.013.
